# Markers of arterial stiffness and urinary metabolomics in young adults with early cardiovascular risk: the African-PREDICT study

**DOI:** 10.1007/s11306-023-01987-y

**Published:** 2023-03-29

**Authors:** Wessel L. du Toit, Ruan Kruger, Lebo F. Gafane-Matemane, Aletta E. Schutte, Roan Louw, Catharina M. C. Mels

**Affiliations:** 1 Hypertension in Africa Research Team (HART), North-West University, Private Bag X6001, Potchefstroom, 2520 South Africa; 2 MRC Research Unit for Hypertension and Cardiovascular Disease, North-West University, Potchefstroom, South Africa; 3 School of Population Health, University of New South Wales, Sydney, Australia; 4 The George Institute for Global Health, Sydney, Australia; 5 Human Metabolomics, North-West University, Potchefstroom Campus, Potchefstroom, South Africa

**Keywords:** Cardiovascular disease, Central systolic blood pressure, Arterial stiffness, Metabolomics, Pulse wave velocity, Risk factors

## Abstract

**Introduction:**

Increased exposure to risk factors in the young and healthy contributes to arterial changes, which may be accompanied by an altered metabolism.

**Objectives:**

To increase our understanding of early metabolic alterations and how they associate with markers of arterial stiffness, we profiled urinary metabolites in young adults with cardiovascular disease (CVD) risk factor(s) and in a control group without CVD risk factors.

**Methods:**

We included healthy black and white women and men (*N* = 1202), aged 20–30 years with a detailed CVD risk factor profile, reflecting obesity, physical inactivity, smoking, excessive alcohol intake, masked hypertension, hyperglycemia, dyslipidemia and low socio-economic status, forming the CVD risk group (*N* = 1036) and the control group (*N* = 166). Markers of arterial stiffness, central systolic blood pressure (BP) and pulse wave velocity were measured. A targeted metabolomics approach was followed by measuring amino acids and acylcarnitines using a liquid chromatography-tandem mass spectrometry method.

**Results:**

In the CVD risk group, central systolic BP (adjusted for age, sex, ethnicity) was negatively associated with histidine, arginine, asparagine, serine, glutamine, dimethylglycine, threonine, GABA, proline, methionine, pyroglutamic acid, aspartic acid, glutamic acid, branched chain amino acids (BCAAs) and butyrylcarnitine (all *P* ≤ 0.048). In the same group, pulse wave velocity (adjusted for age, sex, ethnicity, mean arterial pressure) was negatively associated with histidine, lysine, threonine, 2-aminoadipic acid, BCAAs and aromatic amino acids (AAAs) (all *P* ≤ 0.044). In the control group, central systolic BP was negatively associated with pyroglutamic acid, glutamic acid and dodecanoylcarnitine (all *P* ≤ 0.033).

**Conclusion:**

In a group with increased CVD risk, markers of arterial stiffness were negatively associated with metabolites related to AAA and BCAA as well as energy metabolism and oxidative stress. Our findings may suggest that metabolic adaptations may be at play in response to increased CVD risk to maintain cardiovascular integrity.

**Supplementary Information:**

The online version contains supplementary material available at 10.1007/s11306-023-01987-y.

## Introduction

Exposure to risk factors contributes to premature cardiovascular disease (CVD) development, a global and growing health burden (Rodgers et al., 2019; Stewart et al., 2017; World Health Organisation, 2022a). This predisposition increases as the exposure to CVD related risk factors increase. These may include obesity, physical inactivity, tobacco and alcohol use, elevated blood pressure (BP), hyperglycemia, dyslipidemia and low socio-economic status (SES), or a combination of these risk factors (Banks et al., 2019; Cercato & Fonseca, 2019; Kjeldsen, 2018; Lavie et al., 2019; Matheus et al., 2013; Nelson, 2013; Piano, 2017; Rosengren et al., 2019; Schultz et al., 2018).

In the young and healthy, increased exposure to CVD risk factors may already affect the structure and function of large arteries which may lead to the development of CVD later in life (Bruno et al., 2020; Laurent et al., 2019). These early vascular alterations are reflected by markers of arterial stiffness such as central systolic BP and aortic pulse wave velocity, the gold standard measurement for arterial stiffness (Pauca et al., 2001; Townsend et al., 2015; Van Bortel et al., 2012). Additionally, these early changes may also be accompanied by altered metabolism before the manifestation of clinical CVD (Polonis et al., 2020). Identifying these early metabolic alterations and how they associate with markers of arterial stiffness in the young and healthy may lead to biomarker or pathway discovery normally masked by advanced CVD and age. The discovery of novel biomarkers and related pathways may also lead to the identification of new targets for therapeutic interventions and the development of preventative strategies.

Metabolomics enables the identification of altered pathways or profiles through the quantification of multiple metabolites simultaneously (Barallobre-Barreiro et al., 2013; Kordalewska & Markuszewski, 2015; Lewis et al., 2008; McGarrah et al., 2018; Ussher et al., 2016). In this regard, we have previously demonstrated specific urinary metabolomic profiles and pathways associated with CVD risk, including altered aromatic amino acid (AAA), and branched chain amino acid (BCAA) metabolism, energetics, and oxidative stress within the African Prospective study on Early Detection and Identification of Cardiovascular disease and Hypertension (African-PREDICT) cohort (aged 20–30 years) (du Toit et al., 2022; Mels et al., 2019). However, it remains unclear whether these metabolic pathways associate with vascular alterations in the presence of CVD risk factors.

Therefore, we aimed to investigate the associations between markers of arterial stiffness (central systolic BP and pulse wave velocity) with urinary metabolites in young adults stratified by the presence or absence of CVD risk factors (obesity, physical inactivity, smoking, excessive alcohol intake, masked hypertension, hyperglycemia, dyslipidemia and low SES).

## Methods

### Study design and population

**Fig. 1:**
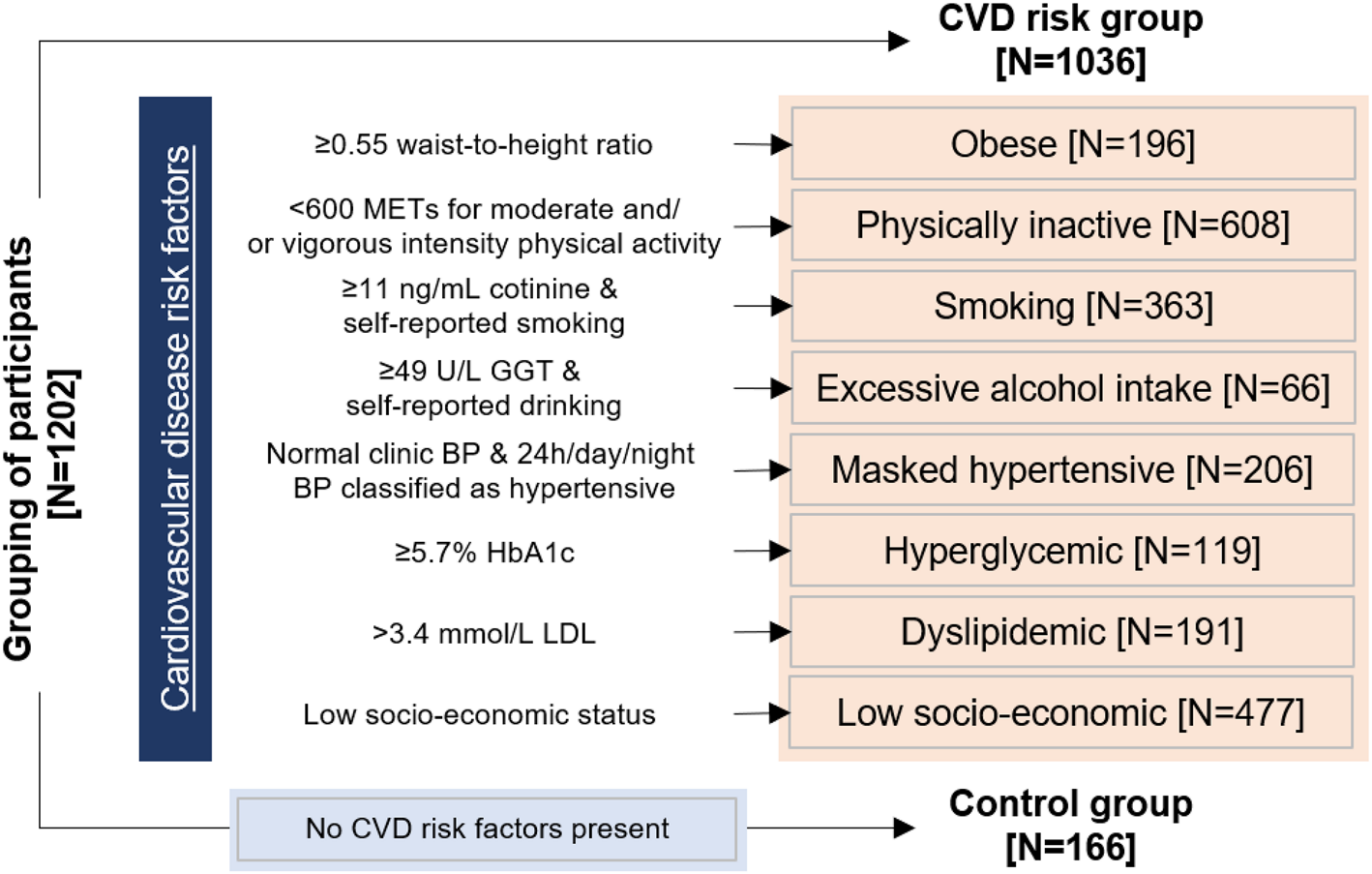
Grouping of participants according to the presence/absence of cardiovascular disease risk factors. Cardiovascular disease risk group criteria and sources: Obese (Amirabdollahian & Haghighatdoost, 2018; Yoo, 2016)—≥ 0.55 waist-to-height ratio; Physically inactive (Keating et al., 2019; World Health Organisation, 2022b)—< 600 METs for moderate and/or vigorous intensity physical activity; Smoking (Kim, 2016; Raja et al., 2016)—≥ 11 ng/mL cotinine & self-reported smoking; Excessive alcohol intake (Agarwal et al., 2016; Jastrzebska et al., 2016; Puukka et al., 2006)—≥ 49 U/L GGT & self-reported drinking; Masked hypertensive (Anstey et al., 2018)—normal clinic BP & 24 h/day/night BP classified as hypertensive; Hyperglycemic (Sherwani et al., 2016)—≥ 5.7% HbA1c; Dyslipidemic (Nelson, 2013; Pagana et al., 2020)—> 3.4 mmol/L LDL; Low socio-economic (Patro et al., 2012)—low SES. *CVD* cardiovascular disease

This study forms part of the African-PREDICT study. Details of the study were previously published (Schutte et al., 2019). In short, the African-PREDICT study is aimed at investigating early CVD-related pathophysiology by tracking young (aged 20–30 years) apparently healthy black and white adults over time (Schutte et al., 2019). Recruitment of participants were done on a voluntary basis from the North-West Province of South Africa. During screening participants were included if they were normotensive (clinic BP was < 140/90 mmHg) (Mancia et al., 2013), uninfected with the human immunodeficiency virus, not diagnosed with chronic diseases or using medication for chronic diseases (self-reported), not pregnant or lactating (self-reported). This study was approved by the Health Research Ethics Committee of the North-West University (NWU-00411-20-A1) and adhered to the principles set out in the Declaration of Helsinki. All participants provided written informed consent. The full baseline cohort of 1202 young adults stratified by the presence or absence of CVD risk factors (obesity—≥ 0.55 waist-to-height ratio, physical inactivity—< 600 metabolic equivalents (METs) for moderate and/or vigorous intensity physical activity, smoking—≥ 11 ng/mL cotinine and self-reported smoking, excessive alcohol intake—≥ 49 U/L gamma-glutamyl transferase (GGT) and self-reported drinking, masked hypertension—normal clinic BP and 24 h/day/night BP classified as hypertensive, hyperglycemia—≥ 5.7% glycated haemoglobin (HbA1c), dyslipidemia—> 3.4 mmol/L low density lipoprotein cholesterol (LDL) and low SES) were cross-sectionally analysed (Fig. 1).

### Questionnaire data

Demographic data were collected using a General Health and Demographic Questionnaire. Data obtained included age, sex, ethnicity, education level, employment information, household income, medication use, smoking and alcohol consumption (used in conjunction with cotinine and GGT respectively, as criteria for the CVD risk group). From the demographic information, SES was calculated using a point system adapted from Kuppuswamy's Socioeconomic Status Scale 2010 (Patro et al., 2012) for a South African environment. Participants were scored in three categories: skill level (classified according to the South African Standard Classification of Occupation), education and income. These three factors were used to categorise participants into socio-economic classes (low, middle, high) and used as criteria for the CVD risk group. Furthermore, a SES score was determined.

Physical activity data were collected using the Global Physical Activity Questionnaire. Data obtained included sedentary behaviour, moderate and vigorous intensity physical activity. From the physical activity information, the METs were calculated, where one MET is defined as the energy cost of sitting quietly and is equivalent to a caloric consumption of 1 kCal/kg/hour; 4 METs is assigned to moderate intensity physical activity and 8 METs is assigned to vigorous intensity physical activity (World Health Organisation, 2022b). The METs were used as criteria for the CVD risk group (< 600 METs for moderate and/or vigorous intensity physical activity—physically inactive) (Keating et al., 2019; World Health Organisation, 2022b). The average of the three variables was used in this study.

Dietary data were collected using a 24 h dietary recall questionnaire. The questionnaire was completed three times, once on site and twice within 7 days (Steinfeldt et al., 2013). Participants answered the questionnaire using a standardised dietary collection kit containing example pictures, packages, measurement tools and food models. Data obtained included protein intake that were coded according to the South African Medical Research Council’s (SAMRC) food composition tables (Wolmarans et al, 2010) and the SAMRC’s food quantities manual (Langenhoven et al., 1991) in grams. Protein intake were used to adjust the metabolomics data, since this may influence amino acid concentrations in the body (Wu, 2016).

### Anthropometric measurements

Anthropometric measurements were taken in accordance with the guidelines of the International Society for the Advancement of Kinanthropometry (International Society for the Advancement of Kinanthropometry, 2001) to obtain height (m), determined by the SECA 213 Portable Stadiometer (SECA, Hamburg, Germany), weight (kg), using the SECA 813 Electronic Scales (SECA, Hamburg, Germany) and waist circumference (cm), using the Lufkin Steel Anthropometric Tape (W606 PM; Lufkin, Apex, USA). Body mass index (BMI) (weight (kg)/height(m^2^)) and waist-to-height ratio were then calculated. Waist-to-height ratio were used as criteria for the CVD risk group (≥ 0.55 waist-to-height ratio—obese) (Amirabdollahian & Haghighatdoost, 2018; Yoo, 2016).

### Cardiovascular measurements

Clinic BP measurements were obtained using the Dinamap Procare 100 Vital Signs Monitor (GE Medical Systems, Milwaukee, USA) (Reinders et al., 2006) apparatus with appropriately sized cuffs and the participant in the upright sitting position. Participants were requested to rest for a 5 min period before and between each measurement and not to have exercised, smoked or eaten for the last 30 min prior to commencement of the measurements. Measurements were taken in duplicate at the left and right brachial artery. The mean of the second measurements at the right and left arm were used to calculate mean arterial pressure.

Ambulatory BP measurements were obtained over 24 h using the Card(X)plore (Meditech, Budapest, Hungary) apparatus with appropriately sized cuffs. The device measured BP in 30 min intervals during daytime (6 a.m. to 10 p.m.) and hourly during the night (10 p.m. to 6 a.m.). The mean successful inflation rate over the 24 h period was 88%. Furthermore, ambulatory BP together with clinic BP measurements were used to identify participants with masked hypertension (normal clinic BP and 24 h (SBP ≥ 130 mmHg and/or DBP ≥ 80 mmHg) /day (SBP ≥ 135 mmHg and/or DBP ≥ 85 mmHg) /night (SBP ≥ 120 mmHg and/or DBP ≥ 70 mmHg) BP classified as hypertensive) which was used as criteria for the CVD risk group (Anstey et al., 2018).

Central systolic BP and pulse wave velocity were obtained using the SphygmoCor® XCEL device (AtCor Medical Pty. Ltd., Sydney, Australia) (Pauca et al., 2001; Townsend et al., 2015; Van Bortel et al., 2012). Participants were requested to be in a supine relaxed position for approximately 5 min before the measurement commenced. Central systolic BP (pulse wave analysis) was determined by placing a brachial cuff on the right upper arm. Pulse wave velocity was determined by locating the right carotid artery, identifying the strongest pulse point though palpation and measuring it with a tonometer. The femoral pulse was measured using a femoral cuff placed around the thigh, while 80% of the distance between the carotid pulse point and upper femoral cuff was calculated and used to measure pulse wave velocity (transit-distance method). Measurements were taken in duplicate. The mean of the measurements was used in this study.

### Biochemical analyses

Blood and spot urine samples were obtained by a registered nurse from fasted participants. The biological samples were immediately prepared and aliquoted into cryovials and stored at -80ºC until analysis. The Cobas Integra® 400 plus (Roche, Basel, Switzerland) were used to analyse GGT, the lipid profile (total cholesterol, high-density lipoprotein cholesterol, LDL and triglycerides) and C-reactive protein in serum samples. Glucose levels in sodium fluoride plasma samples and HbA1c in EDTA whole blood samples were also analysed using the Cobas Integra® 400 plus (Roche, Basel, Switzerland). Cotinine was analysed from serum samples using the Immulite (Siemens, Erlangen, Germany) apparatus. Serum peroxides as a measure of reactive oxygen species (ROS), were analysed in serum samples (Hayashi et al., 2007) using Synergy H4 hybrid microplate reader (BioTek, Winooski, VT, USA). Metabolomics data (30 amino acids and 9 acylcarnities) were analysed using a liquid chromatography-tandem mass spectrometry method on an Agilent^©^ system (1200 series LC front end coupled to a 6410 series triple quadrupole mass analyser) with electrospray ionisation source operated in positive ionisation mode. Details of this method were previously published (du Toit et al., 2022). In short, urine samples were randomised to be prepared and analysed in batches of 20 samples per batch, together with 3 quality control urine samples and an additional in-house standard mixture (consisting of all analysed metabolites), to monitor data integrity. Sample preparation started with defrosting urine samples overnight, after which an isotope mixture containing various amino acid and acylcarnities isotopes were added to a predetermined volume of urine. Thereafter the urine samples were further processed and stored (− 80 °C) until analysis. Before analysis the samples were again defrosted and processed for metabolite separation using a Zorbax SB-Aq 80 Å StableBond column (Agilent^©^, 2.1 mm × 100 mm × 1.8 μ; cat# 828700-914) and Zorbax Eclipse Plus C18 guard column (Agilent^©^, 2.1 mm × 5 mm, 1.8 μm, cat# 821725-901) with specific run order times and parameters. Regarding data prepossessing, a peak intensity filter was applied to remove features with areas below the limit of quantification (LOQ cut-off of area < 750). Metabolomics data were then normalised to the added isotope internal standards. Furthermore, spectral data matrices were individually inspected for each batch to ensure good data quality. Altogether, the data proved to be good quality with no batch effects visible. Biochemical variables used as criteria for the CVD risk group include, GGT (≥ 49 U/L GGT and self-reported alcohol use—excessive alcohol intake) (Agarwal et al., 2016; Jastrzebska et al., 2016; Puukka et al., 2006), LDL (> 3.4 mmol/L LDL—dyslipidemic) (Nelson, 2013; Pagana et al., 2020), HbA1c (≥ 5.7% HbA1c—hyperglycemic) (Sherwani et al., 2016) and cotinine (≥ 11 ng/mL cotinine and self-reported tobacco use—smoking) (Kim, 2016; Raja et al., 2016).

### Statistical analyses

Statistical analyses were performed with IBM®, SPSS® version 27 (IBM Corporation, Armonk, New York). Variables were tested for normality and logarithmically transformed if skewed. Logged variables included physical activity, cotinine, GGT, triglycerides, C-reactive protein, ROS, protein intake and the metabolomics data. Data is reported as mean (normally distributed variables) or geometric mean (logarithmically transformed variables) with 95% confidence intervals. Grouping of participants were performed according to the presence of CVD risk factor(s), forming the CVD risk group and the control group (Fig. 1). The characteristics between the control and CVD risk group were compared using the Chi-square test to compare categorical variables and ANCOVA to compare continuous variables. In the ANCOVAs, adjustment was made for sex and ethnicity, for pulse wave velocity further adjustment was made using mean arterial pressure, and for the metabolomics data further adjustment was made for protein intake (as part of a sensitivity analysis). *P*-values for comparing metabolomics data between the control and CVD risk group were adjusted for multiple comparisons to lower the false discovery rate using the Benjamini–Hochberg procedure (*q*-value). Multivariable adjusted regression analyses were performed to determine associations between central systolic BP and pulse wave velocity with the metabolomics data in the control and CVD risk group. The basic model included age, sex and ethnicity with additional adjustment for pulse wave velocity using mean arterial pressure. Furthermore, as part of the multiple regression analysis, a sensitivity analysis was performed in the control and CVD risk group taking into consideration protein intake as a covariate. The data underlying this article are available in the article and in its online supplementary material.

## Results

The demographics and cardiovascular risk factor comparison between the control and CVD risk group are shown in Table 1. As expected, the CVD risk group showed a worse CVD risk profile compared to the control group (all *P* ≤ 0.003). Markers of arterial stiffness revealed higher central systolic BP in the CVD risk group compared to the control group (*P* = 0.003), with no difference in pulse wave velocity between the groups (*P* = 0.858). Markers of inflammation (C-reactive protein) and oxidative stress (serum peroxides) were also higher in the CVD risk group compared to the control group (*P* ≤ 0.009). The metabolomics comparison (Supplementary Table 1) indicated higher creatine, tyrosine and phenylalanine in the CVD risk group compared to the control group (all *P* ≤ 0.044). We additionally adjusted the metabolomics data for protein intake, since this may influence amino acid levels in the body (Wu, 2016). After the adjustment phenylalanine lost significance (Supplementary Table 2). Furthermore, after performing the Benjamini–Hochberg procedure the differences between all metabolites lost statistical significance.

**Table 1:** Comparisons of demographics and cardiovascular risk factors between the control and cardiovascular disease risk group

	Control group	CVD risk group	*P*-value
*N*	166	1036	
Age (years)	24.8 (24.3; 25.3)	24.5 (24.3; 24.7)	0.238
Sex, female [n (%)]	108 (65.1)	516 (49.8)	** < 0.001**
Ethnicity, black [n (%)]	39 (23.5)	567 (54.7)	** < 0.001**
Cardiovascular disease risk factors			
Weight (kg)	65.4 (62.9; 67.9)	72.2 (71.2; 73.1)	** < 0.001**
Waist circumference (cm)	74.7 (72.9; 76.5)	81.0 (80.3; 81.7)	** < 0.001**
Height (m)	1.69 (1.68; 1.70)	1.68 (1.68; 1.69)	0.758
Body mass index (kg/m^2^)	23.0 (22.2; 23.9)	25.4 (25.1; 25.7)	** < 0.001**
*Waist-to-height ratio*	*0.44 (0.43; 0.45)*	*0.48 (0.48; 0.49)*	** < *0.001***
*Physical activity (kCal/kg/day)*	*271 (240; 309)*	*206 (195; 219)*	** < *0.001***
*Cotinine (ng/ml)*	*1.15 (0.83; 1.58)*	*4.32 (3.80; 4.90)*	** < *0.001***
*Self-reported smoking [n (%)]*	*0 (0)*	*286 (27.6)*	** < *0.001***
*γ-glutamyl transferase (U/L)*	*13.1 (12.0; 14.5)*	*19.2 (18.6; 20.0)*	** < *0.001***
*Self-reported alcohol use [n (%)]*	*69 (41.6)*	*597 (58.1)*	** < *0.001***
24 h Systolic BP (mmHg)	114 (113; 115)	117 (117; 118)	** < 0.001**
24 h Diastolic BP (mmHg)	67 (66; 68)	69 (69; 69)	** < 0.001**
MAP (mmHg)	93 (92; 94)	94 (94; 95)	0.128
Central systolic BP (mmHg)	106 (105; 108)	108 (108; 109)	**0.003**
Pulse wave velocity (m/s*)	6.33 (6.20; 6.46)	6.34 (6.29; 6.39)	0.858
*Masked hypertensive, [n (%)]*	*0 (0)*	*206 (20.1)*	** < *0.001***
*HbA1c (%)*	*5.26 (5.21; 5.30)*	*5.33 (5.31; 5.35)*	***0.003***
Glucose (mmol/L)	3.85 (3.69; 4.02)	4.13 (4.07; 4.19)	**0.002**
Total cholesterol (mmol/L)	3.29 (3.12; 3.47)	3.83 (3.76; 3.90)	** < 0.001**
HDL cholesterol (mmol/L)	1.17 (1.11; 1.23)	1.16 (1.13; 1.18)	0.734
*LDL cholesterol (mmol/L)*	*2.03 (1.88; 2.17)*	*2.51 (2.45; 2.57)*	** < *0.001***
Triglycerides (mmol/L)	0.56 (0.52; 0.62)	0.75 (0.72; 0.78)	** < 0.001**
C-reactive protein (mg/L)	0.59 (0.47; 0.74)	0.95 (0.87; 1.05)	** < 0.001**
ROS, mg/L H_2_O_2_	34.3 (30.9; 38.0)	39.4 (38.0; 40.7)	**0.009**
*Socio-economic status score*	*23.5 (22.7; 24.3)*	*20.2 (19.9; 20.5)*	** < *0.001***
Protein intake (g)	69.7 (64.6; 74.1)	66.5 (64.6; 67.6)	0.224

Tests used: Chi-square tests and ANCOVAs (adjusted for sex and ethnicity with additional adjustment for *pulse wave velocity using mean arterial pressure). Data are presented as mean or geometric mean with 95% confidence intervals. Bold values denote *P* ≤ 0.05. Cardiovascular disease risk group criteria: Obese—≥ 0.55 waist-to-height ratio; Physically inactive—< 600 METs for moderate and/or vigorous intensity physical activity; Smoking—≥ 11 ng/mL cotinine AND self-reported smoking; Excessive alcohol intake—≥ 49 U/L GGT and self-reported drinking; Masked hypertensive—normal clinic BP and 24 h/day/night BP classified as hypertensive; Hyperglycemic—≥ 5.7% HbA1c; Dyslipidemic—> 3.4 mmol/L LDL; Low socio-economic—low SES

*γ* gamma, *MAP* mean arterial pressure, *BP* blood pressure, *HbA1c* glycated haemoglobin, *HDL* high-density lipoprotein, *LDL* low-density lipoprotein, *ROS* reactive oxygen species, *CVD* cardiovascular disease

**Fig. 2:**
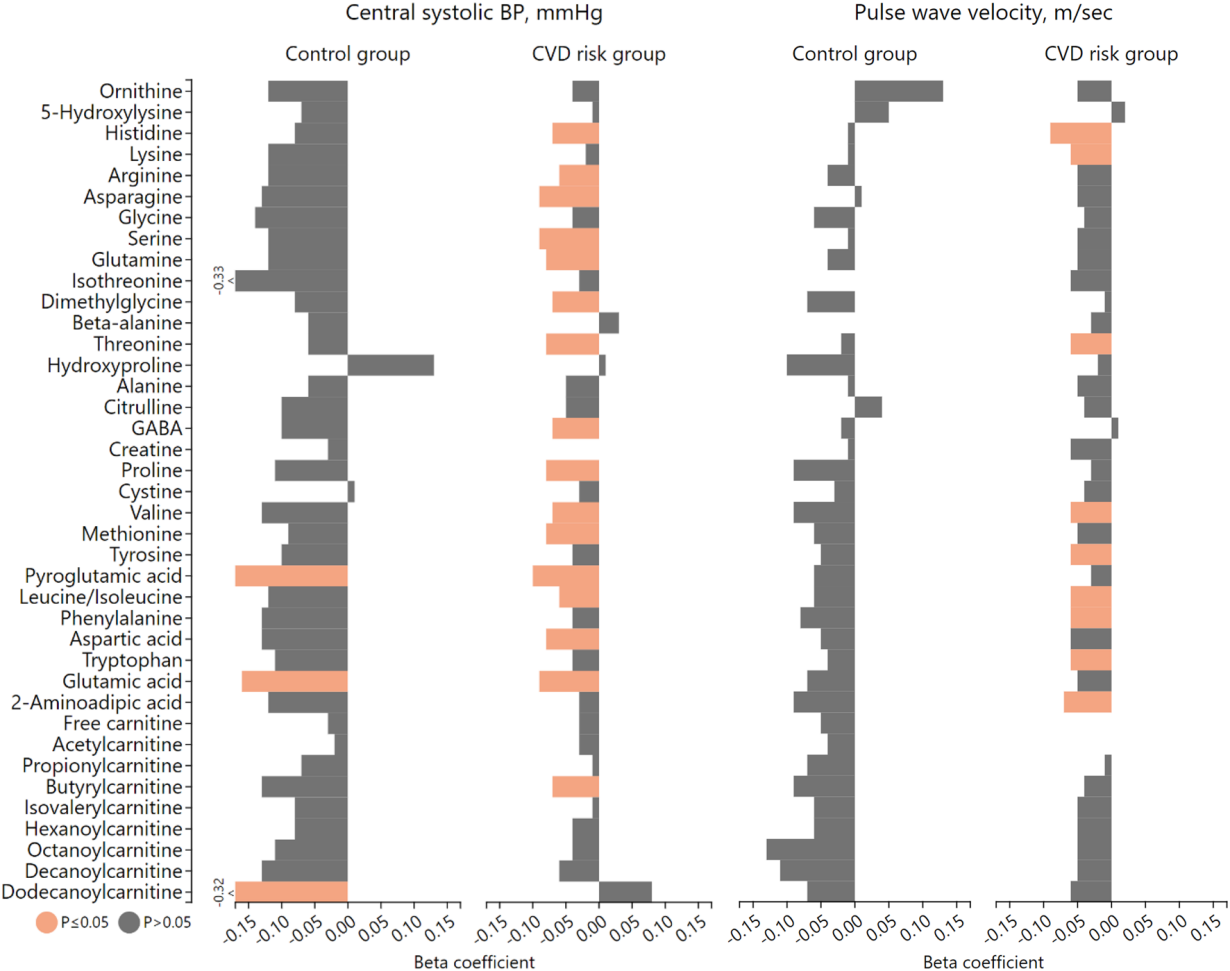
Multi-variable adjusted regression analysis with central systolic blood pressure or pulse wave velocity as the dependent variable, with the metabolomics data in the control and cardiovascular disease risk group. Test used: Multiple linear regressions. β coefficients are presented—separate models. Central systolic BP, adjusted for age, sex, ethnicity; pulse wave velocity, adjusted for age, sex, ethnicity, mean arterial pressure. Cardiovascular disease risk group criteria: Obese—≥ 0.55 waist-to-height ratio; Physically inactive—< 600 METs for moderate and/or vigorous intensity physical activity; Smoking—≥ 11 ng/mL cotinine & self-reported smoking; Excessive alcohol intake—≥ 49 U/L GGT & self-reported drinking; Masked hypertensive—normal clinic BP & 24 h/day/night BP classified as hypertensive; Hyperglycemic—≥ 5.7% HbA1c; Dyslipidemic—> 3.4 mmol/L LDL; Low socio-economic—low SES. Metabolite concentration expressed as arbitrary units. *BP* blood pressure, *CVD* cardiovascular disease

Using adjusted regression models, we determined whether central systolic BP (adjusted for age, sex and ethnicity) and pulse wave velocity (adjusted for age, sex, ethnicity and mean arterial pressure) were associated with the metabolomics data in the control and CVD risk group (Fig. 2 and Supplementary Table 3A-D). In the CVD risk group, we found negative associations between central systolic BP and histidine, arginine, asparagine, serine, glutamine, dimethylglycine, threonine, GABA, proline, valine, methionine, pyroglutamic acid, leucine/isoleucine, aspartic acid, glutamic acid and butyrylcarnitine (all *P* ≤ 0.048). Additionally, we found negative associations between pulse wave velocity and histidine, lysine, threonine, valine, tyrosine, leucine/isoleucine, phenylalanine, tryptophan and 2-aminoadipic acid (all *P* ≤ 0.044). In the control group, negative associations were found between central systolic BP and pyroglutamic acid, glutamic acid and dodecanoylcarnitine (all *P* ≤ 0.033).

### Sensitivity analysis

We additionally performed a sensitivity analysis in the control and CVD risk group and included protein intake as an additional covariate in the multiple regression model (Supplementary Table 4A–D). This was done since protein intake may influence amino acid levels in the body (Wu, 2016). After the additional adjustment for protein intake the associations between central systolic BP and arginine (*P* = 0.052), GABA (*P* = 0.052) and leucine/isoleucine (*P* = 0.063) in the CVD risk group lost significance. All the other associations remained significant.

## Discussion

Comparing the metabolite concentrations between the control and CVD risk group revealed no statistically significant differences, this could be explained in part by the young age and apparent healthy nature of the research participants. However, when considering the multi-variate adjusted regression analysis, negative associations were found between central systolic BP and pulse wave velocity with metabolites associated with AAA and BCAA metabolism, energy metabolism and oxidative stress in a study population consisting of young apparently healthy black and white men and women with one or more CVD risk factors. In contrast to other metabolomic studies, which were mostly conducted in aged adults and in those with overt vascular complications and established CVD, such as arterial stiffness, coronary artery disease, peripheral artery disease and hypertension (Koh et al., 2018; Li et al., 2019; Menni et al., 2015; Paapstel et al., 2016; Polonis et al., 2020; Zagura et al., 2015), our findings highlight the early metabolic changes associated with markers of arterial stiffness in individuals at risk for the development of CVD.

### Aromatic and branched chain amino acid metabolism

**Fig. 3:**
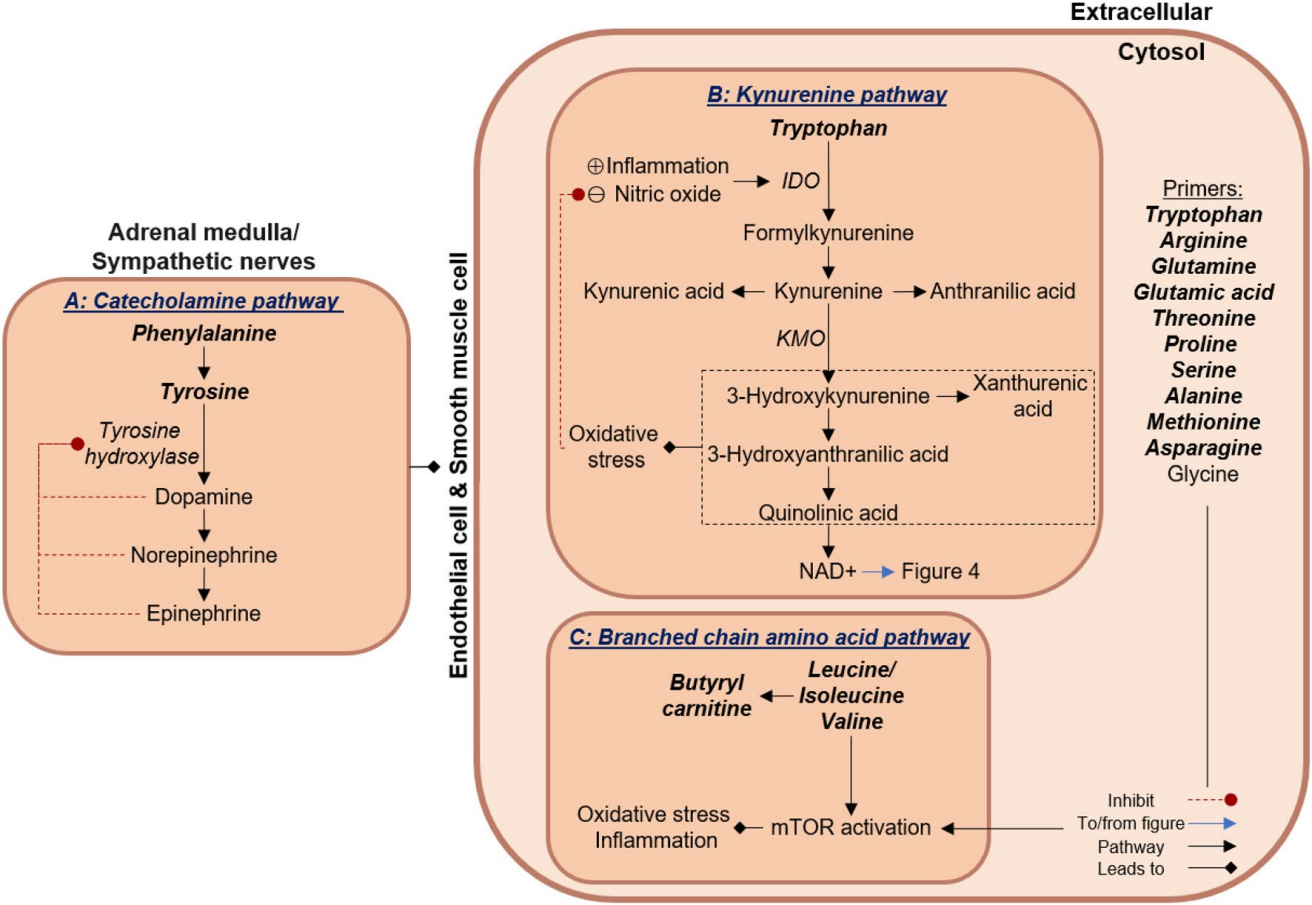
Markers of arterial stiffness relate to altered aromatic amino acid and branched chain amino acid metabolism within the cardiovascular disease risk group. Within the CVD risk group, central systolic BP and pulse wave velocity showed inverse associations with metabolites linked to AAA and BCAA metabolism. Phenylalanine and tyrosine feed into the catecholamine pathway, producing dopamine, norepinephrine and epinephrine which is implicated in vascular tone. Tryptophan is metabolised through the kynurenine pathway which is induced by inflammation and when nitric oxide is unable to inhibit this pathway. Kynurenine is implicated in vascular tone; the downstream metabolites is linked to oxidative stress and inflammation. The BCAAs, through mTOR activation, preserve cardiovascular integrity and enable cardiovascular adaptation; increased activation causes oxidative stress and inflammation. Metabolites associated with markers of arterial stiffness are indicated in bold and italic. *IDO* indoleamine-2,3-dioxygenase, *KMO* kynurenine 3-monooxygenase; *NAD+* nicotinamide adenine dinucleotide, *mTOR* mammalian target of rapamycin

In the CVD risk group, inverse associations were found between pulse wave velocity and the AAAs, phenylalanine and tyrosine. This is in contrast to a previous metabolomic study conducted in aged men (66 years) with peripheral artery disease in which positive associations were found between pulse wave velocity and the AAAs (Zagura et al., 2015). Phenylalanine, an essential amino acid, is metabolised to tyrosine, a precursor for the synthesis of catecholamines such as dopamine, norepinephrine, and epinephrine (Motiejunaite et al., 2021) (Fig. 3A). This metabolic pathway is controlled by the rate limiting enzyme, tyrosine hydroxylase, which is regulated by feedback inhibition by the respective catecholamines (Dickson & Briggs, 2013; Motiejunaite et al., 2021). These catecholamines activate different adrenergic receptor(s), which lead to specific effects depending on the type of receptors activated, the location of receptors (blood vessels, heart, brain) and signaling cascades activated (Motiejunaite et al., 2021; Sorriento et al., 2011). In a young study population without CVD, but with increased CVD risk, the inverse associations found between pulse wave velocity with phenylalanine and tyrosine may suggest that the catecholamine synthesis, and hence binding to the adrenergic receptors are decreased via a negative feedback mechanism to maintain vasodilation. The negative feedback mechanism may thus result in the higher phenylalanine and tyrosine levels observed in this group. When considering that pulse wave velocity was similar in both groups and within normal ranges (Van Bortel et al., 2012) this may be an adaptive response to maintain the elasticity of the central arteries despite the increased CVD risk.

Tryptophan, an essential amino acid, was also inversely associated with pulse wave velocity in the CVD risk group. Uncontrolled tryptophan metabolism has been associated with numerous vascular complications and CVD such as atherosclerosis, endothelial dysfunction, heart disease and hypertension (Ramprasath et al., 2021; Song et al., 2017). Tryptophan metabolism occurs mainly via the kynurenine pathway (> 95% of tryptophan metabolism), where tryptophan is converted to formylkynurenine by two rate-limiting enzymes, tryptophan 2,3-dioxygenase (TDO) (hepatic) and indoleamine-2,3-dioxygenase (IDO) (extrahepatic, such as endothelial and vascular smooth muscle cells) (Badawy, 2019; Ramprasath et al., 2021; Song et al., 2017) (Fig. 3B). Usually, TDO governs basal tryptophan metabolism, while IDO increases tryptophan metabolism under inflammatory and oxidative conditions (decreased nitric oxide (NO)) (Badawy, 2019; Ramprasath et al., 2021; Song et al., 2017). In a young study population without CVD, but with increased CVD risk, ROS levels and the inflammatory marker C-reactive protein were higher (but still within normal ranges) (Forman et al., 2016; Pagana et al., 2020) when compared to the control group. Since kynurenine was shown to induce arterial relaxation (Badawy, 2019; Ramprasath et al., 2021; Song et al., 2017), we hypothesise that the inverse association between pulse wave velocity and tryptophan, may reflect an activated kynurenine pathway with consequent increased vasodilation to maintain vascular tone at physiological levels. However, increased flux through the one branch of the kynurenine pathway i.e. metabolism of kynurenine by kynurenine 3-monooxygenase (KMO) to ultimately produce nicotinamide adenine dinucleotide (NAD +), may lead to the formation of pro-oxidative and pro-inflammatory metabolites such as 3-hydroxykynurenine, xanthurenic acid, 3-hydroxyanthranilic acid and quinolinic acid (Badawy, 2019; Ramprasath et al., 2021; Song et al., 2017). The accumulation of these metabolites may result in a vicious cycle of IDO activation and consequently vascular complications and CVD (Badawy, 2019; Ramprasath et al., 2021; Song et al., 2017). Therefore, inhibiting KMO activity might be beneficial in CVD with increased inflammation (Badawy, 2019; Phillips et al., 2019). This in turn will result in increased kynurenine and downstream metabolites, kynurenic acid and anthranilic acid (through different branches) which has antioxidant properties (Francisco-Marquez et al., 2016; Lugo-Huitrón et al., 2011).

Furthermore, in this group with CVD risk, both central systolic BP and pulse wave velocity associated inversely with the BCAAs (leucine/isoleucine and valine), and with butyrylcarnitine (central systolic BP only), a product of BCAA metabolism (Fig. 3C). Branched-chain amino acids, along with other nutrient signals such as insulin, lead to mammalian target of rapamycin (mTOR) activation (Laplante & Sabatini, 2012; Sciarretta et al., 2018). Physiologically, this activation is essential for the preservation of cardiovascular integrity and enable cardiovascular adaptation to mechanical stress (Sciarretta et al., 2018). In a young study population without CVD, but with increased CVD risk, central and 24 h BP were higher when compared to the control group. We therefore hypothesise that the inverse association between central systolic BP, pulse wave velocity and the BCAAs, may reflect mTOR activation with consequent cardiovascular adaptation to maintain cardiovascular integrity. However, deregulation of mTOR have also been associated with CVD (Chong et al., 2011; Dyachok et al., 2016; Laplante & Sabatini, 2012; Sciarretta et al., 2018; Zhenyukh et al., 2018), it is therefore essential that activation of mTOR remain in a compensatory state, preventing deregulated activation and the pathological consequences leading to CVD.

### Cardiovascular energy metabolism

In the CVD risk group, inverse associations of central systolic BP and pulse wave velocity were found with several amino acids related to energy producing pathways such as glycolysis (the primary energy-producing mechanism in endothelial cells) and the citric acid cycle including, histidine, threonine, BCAAs (associated with central systolic BP and pulse wave velocity); serine, glutamic acid, glutamine, dimethylglycine, methionine, arginine, aspartic acid, asparagine, proline, GABA (associated with central systolic BP); AAAs, lysine, and 2-aminoadipic acid (associated with pulse wave velocity).

**Fig. 4:**
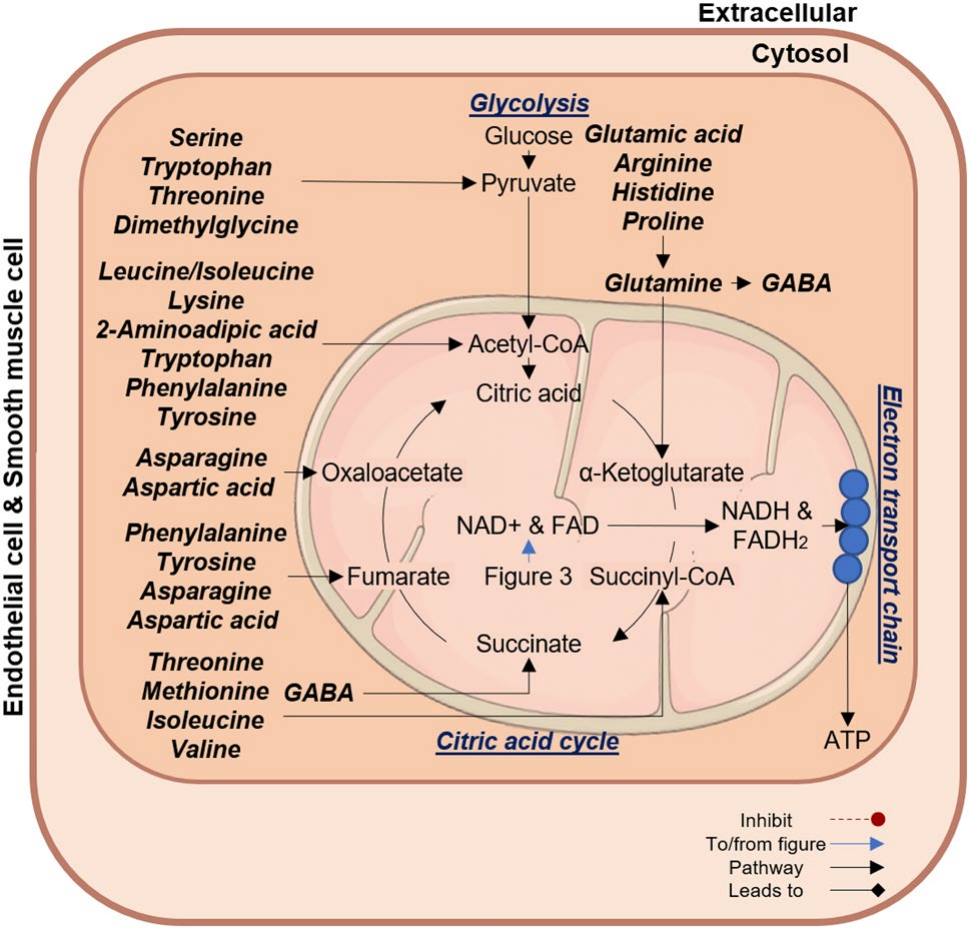
Markers of arterial stiffness relate to altered energetics within the cardiovascular disease risk group. Within the CVD risk group, central systolic BP and pulse wave velocity showed inverse associations with metabolites linked to glycolysis and the citric acid cycle. Glycolysis is the primary energy-producing mechanism in the vasculature. This pathway uses amino acids as substrate to generate ATP. Metabolites associated with markers of arterial stiffness are indicated in bold and italic. *ATP* adenosine triphosphate, *NAD+*  nicotinamide adenine dinucleotide, *FAD* flavin adenine dinucleotide

All of these amino acids feed into glycolysis or the citric acid cycle on different levels such as pyruvate, acetyl-CoA or various citric acid cycle intermediates to produce the reducing agents flavin adenine dinucleotide (FADH_2_) and NADH, which subsequently enters the electron transport chain to generate adenosine triphosphate (ATP) (Akram, 2014) (Fig. 4). Although ATP is considered the major energy source in the cell, it is also a potent extracellular signalling molecule which can be released from all major cell types in the vessel wall to act as an autocrine or paracrine (Ralevic & Dunn, 2015; Wu et al., 2022). Binding of ATP to the different purinergic receptors causes an overall influx of Ca^2+^ into vascular smooth muscle cells or endothelial cells with consequent activation of endothelial NO synthase and NO production (Ralevic & Dunn, 2015; Wu et al., 2022). In a young study population without CVD, but with increased CVD risk, the inverse associations found between central systolic BP, pulse wave velocity and various amino acids related to ATP production, we hypothesise that more amino acids may be made available for ATP production and the consequent binding to the receptors in an attempt to maintain vascular tone in the presence of CVD risk factors. However, if the CVD risk persist over time, this may lead to detrimental consequences as chronic increases in ATP may potentiate hypertension and atherosclerosis (Huang et al., 2021; Ralevic & Dunn, 2015; Wu et al., 2022; Zhao et al., 2019). Therefore, it is essential that the extracellular concentration of this nucleotide is tightly regulated.

### Oxidative stress

**Fig. 5:**
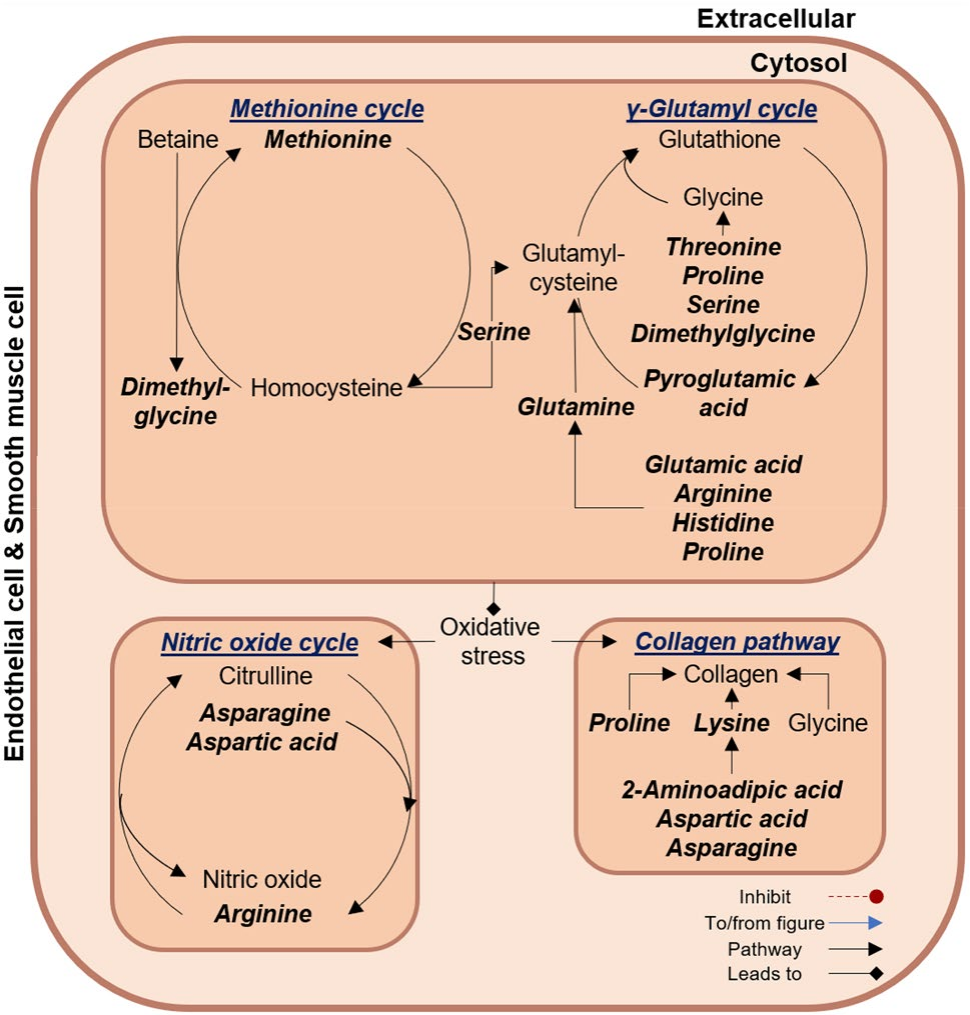
Markers of arterial stiffness relate to oxidative stress within the cardiovascular disease risk group. Within the CVD risk group, central systolic BP and pulse wave velocity showed inverse associations with metabolites linked to the methionine and y-glutamyl cycle. These pathways are important in producing the antioxidant glutathione to alleviate oxidative stress. Increased oxidative stress will in turn affect the NO bioavailability and cause vascular damage (fibrosis). Metabolites associated with markers of arterial stiffness are indicated in bold and italic

Several metabolites which may serve as precursors for the y-glutamyl cycle, were inversely associated with central systolic BP and pulse wave velocity in the CVD risk group. These included histidine, threonine (associated with central systolic BP and pulse wave velocity); serine, proline, arginine, glutamic acid, glutamine, methionine, dimethylglycine and pyroglutamic acid (associated with central systolic BP) (Fig. 5).

The y-glutamyl cycle produces glutathione from glutamine (with histidine, arginine, proline, and glutamic acid as precursors), cysteine (with methionine and serine as precursors) and glycine (with threonine, proline, serine, and dimethylglycine as precursors) (Durante, 2019; Lushchak, 2012). Glutathione is important in maintaining a healthy redox state by serving as an antioxidant (Durante, 2019; Holeček, 2020; Lushchak, 2012). Our finding of an inverse association between markers of arterial stiffness with pyroglutamic acid levels, an intermediate of the y-glutamyl cycle, along with the precursors for glutathione, may suggest that more amino acids may be made available to produce glutathione (Durante, 2019; Gueta et al., 2020; Lushchak, 2012; Venkataraman et al., 2019), possibly due to increased oxidative stress. As stated above, we indicated elevated ROS levels in the CVD risk group. Additionally, some of the precursors for the y-glutamyl cycle such as methionine and cysteine may also contribute to an oxidative environment, through pro-inflammatory, pro-oxidant and pro-atherogenic effects (Fu et al., 2019; Ganguly & Alam, 2015; Garlick, 2006; Rehman et al., 2020; Xiao et al., 2015). This oxidative environment may decrease the bioavailability of NO with consequent vascular damage (fibrosis) (Cyr et al., 2020; Stakos et al., 2010; Zhao et al., 2015).

### Strengths and limitations

The cross-sectional design of this study prevents us from inferring causal relationships. A major strength of our study was that we focused on high-level metabolomics data in a young apparently healthy population from African and European descent without CVD, thereby minimizing the influence of age and disease on the metabolism. We are also among the first to present this type of findings in a multi-ethnic cohort, which is especially limited in Africa.

## Conclusion

In conclusion, in a young study population without CVD, but with increased CVD risk, markers of arterial stiffness were inversely associated with metabolites linked to AAA and BCAA metabolism, energy metabolism and oxidative stress. These pathways may be regulated as an adaptive response to maintain cardiovascular integrity in the presence of CVD risk factors. However, with continued exposure to CVD risk factors these pathways may become dysregulated as previously implicated in CVD.

### Recommendations

Longitudinal studies investigating the associations between markers of arterial stiffness and urinary metabolites. Metabolomic studies focusing on individual CVD risk factors and how this translates to markers of arterial stiffness. Furthermore, future studies to test the associations found in this hypothesis-generating study by investigating the relevant pathways in a targeted manner.

## Supplementary Information

Below is the link to the electronic supplementary material.

Supplementary file1 (DOCX 149 kb)
