# Supplementary material for: Markers of arterial stiffness and urinary metabolomics in young adults with early cardiovascular risk: the African-PREDICT study
Source: Metabolomics. 2023 Mar 29;19(4):28. doi: 10.1007/s11306-023-01987-y (PMC10060307; doi:10.1007/s11306-023-01987-y)
Supplement: Supplementary file 1 — Supplementary file1 (DOCX 149 kb) [file 11306_2023_1987_MOESM1_ESM.docx]

**Markers of arterial stiffness** **and urinary metabolomics in young adults with early cardiovascular risk: The African-PREDICT study**

Wessel L. du Toit^1^, Ruan Kruger^1,2^, Lebo F. Gafane-Matemane^1,2^_,_ Aletta E. Schutte^1,2,3^, Roan Louw^4^, Catharina M.C. Mels^1,2^

^1^*Hypertension in Africa Research Team (HART), North-West University, Potchefstroom, South Africa.*

^2^*MRC Research Unit for Hypertension and Cardiovascular Disease, North-West University, Potchefstroom, South* *Africa.*

*^3^School of Population Health, University of New South Wales; The George Institute for Global Health, Sydney, Australia.*

*^4^Human Metabolomics, North-West University, Potchefstroom Campus, Potchefstroom, South Africa.*

**Correspondence:**

Prof. Carina Mels

Hypertension in Africa Research Team (HART)

North-West University

Private Bag X6001

Potchefstroom

2520

South Africa

Tel: +27 18 299 1983

Fax: +27 18 285 2432

E-mail: [carina.mels@nwu.ac.za](mailto:carina.mels@nwu.ac.za)

Journal: *Metabolomics*

**Supplementary Table 1. Metabolomic data of control and cardiovascular disease risk group**

| ***Metabolomic data*** | **Control group (N=166)** | **CVD risk group (N=1036)** | **P-value** | **q-value** |
| --- | --- | --- | --- | --- |
| Ornithine, AU | 8.89 (7.24; 11.0) | 7.48 (6.92; 7.94) | 0.120 | 0.387 |
| 5-Hydroxylysine, AU | 8.99 (7.76; 10.2) | 9.66 (9.12; 10.2) | 0.345 | 0.561 |
| Histidine, AU | 724 (646; 813) | 733 (708; 759) | 0.872 | 0.953 |
| Lysine, AU | 35.8 (31.6; 40.7) | 37.3 (35.5; 38.9) | 0.558 | 0.680 |
| Arginine, AU | 11.8 (10.7; 12.9) | 12.1 (11.8; 12.6) | 0.493 | 0.663 |
| Asparagine, AU | 24.2 (21.9; 26.9) | 24.2 (23.4; 25.1) | 0.945 | 0.953 |
| Glycine, AU | 501 (437; 575) | 527 (501; 550) | 0.490 | 0.663 |
| Serine, AU | 224 (200; 245) | 236 (229; 245) | 0.340 | 0.561 |
| Glutamine, AU | 220 (195; 251) | 236 (224; 245) | 0.341 | 0.561 |
| Isothreonine, AU | 3.28 (2.40; 4.47) | 3.74 (3.39; 4.17) | 0.436 | 0.630 |
| Dimethylglycine, AU | 15.9 (12.6; 20.0) | 16.0 (14.8; 17.4) | 0.953 | 0.953 |
| Beta-alanine, AU | 45.1 (35.5; 57.5) | 51.6 (46.8; 56.2) | 0.290 | 0.548 |
| Threonine, AU | 77.1 (67.6; 87.1) | 81.7 (77.6; 85.1) | 0.396 | 0.594 |
| Hydroxyproline, AU | 3.33 (2.57; 4.27) | 3.51 (3.24; 3.80) | 0.711 | 0.840 |
| Alanine, AU | 223 (195; 257) | 249 (234; 263) | 0.129 | 0.387 |
| Citrulline, AU | 4.66 (4.17; 5.25) | 4.72 (4.47; 4.90) | 0.841 | 0.953 |
| GABA, AU | 0.24 (0.22; 0.27) | 0.26 (0.25; 0.27) | 0.233 | 0.501 |
| Creatine, AU | 16.3 (14.1; 18.6) | 19.5 (18.6; 20.4) | **0.017** | 0.332 |
| Proline, AU | 1.72 (1.55; 1.91) | 1.83 (1.78; 1.91) | 0.241 | 0.501 |
| Cystine, AU | 0.60 (0.54; 0.68) | 0.67 (0.65; 0.71) | 0.086 | 0.351 |
| Valine, AU | 4.68 (4.17; 5.25) | 5.21 (5.01; 5.50) | 0.063 | 0.351 |
| Methionine, AU | 1.13 (1.02; 1.26) | 1.20 (1.15; 1.26) | 0.244 | 0.501 |
| Tyrosine, AU | 32.1 (28.8; 35.5) | 37.9 (36.3; 39.8) | **0.004** | 0.156 |
| Pyroglutamic acid, AU | 23.1 (20.9; 25.7) | 24.8 (24.0; 25.7) | 0.184 | 0.478 |
| Leucine/Isoleucine, AU | 11.9 (10.7; 13.2) | 13.2 (12.6; 13.8) | 0.072 | 0.351 |
| Phenylalanine, AU | 16.3 (14.5; 18.2) | 18.4 (17.8; 19.1) | **0.044** | 0.351 |
| Aspartic acid, AU | 4.55 (4.17; 5.01) | 4.79 (4.68; 5.01) | 0.295 | 0.548 |
| Tryptophan, AU | 1.13 (0.98; 1.29) | 1.30 (1.23; 1.38) | 0.058 | 0.351 |
| Glutamic acid, AU | 2.15 (1.91; 2.40) | 2.38 (2.29; 2.51) | 0.09 | 0.351 |
| 2-Aminoadipic acid, AU | 0.85 (0.74; 0.98) | 0.89 (0.85; 0.93) | 0.542 | 0.680 |
| Free carnitine, AU | 44.4 (39.8; 49.0) | 46.8 (44.7; 49.0) | 0.375 | 0.585 |
| Acetylcarnitine, AU | 11.5 (9.33; 14.5) | 11.4 (10.5; 12.3) | 0.920 | 0.953 |
| Propionylcarnitine, AU | 1.58 (1.32; 1.91) | 1.69 (1.58; 1.82) | 0.524 | 0.680 |
| Butyrylcarnitine, AU | 11.2 (9.77; 12.9) | 11.2 (10.7; 11.8) | 0.941 | 0.953 |
| Isovalerylcarnitine, AU | 0.52 (0.45; 0.59) | 0.58 (0.55; 0.60) | 0.144 | 0.401 |
| Hexanoylcarnitine, AU | 0.06 (0.05; 0.07) | 0.07 (0.06; 0.07) | 0.106 | 0.376 |
| Octanoylcarnitine, AU | 0.31 (0.28; 0.34) | 0.34 (0.32; 0.35) | 0.055 | 0.351 |
| Decanoylcarnitine, AU | 0.18 (0.16; 0.21) | 0.21 (0.19; 0.22) | 0.079 | 0.351 |
| Dodecanoylcarnitine, AU | 0.02 (0.02; 0.03) | 0.02 (0.02; 0.03) | 0.204 | 0.497 |

Test used: ANCOVAs (adjusted for sex and ethnicity). Data are presented as geometric mean with 95% confidence intervals. Benjamini-Hochberg correction (q-value). Bold values denote P/q≤0.05. Cardiovascular disease risk group criteria: Obese - ≥0.55 waist-to-height ratio; Physically inactive - <600 METs for moderate and/or vigorous intensity physical activity; Smoking - ≥11 ng/mL cotinine & self-reported smoking; Excessive alcohol intake - ≥49 U/L GGT & self-reported drinking; Masked hypertensive - normal clinic BP & 24h/day/night BP classified as hypertensive; Hyperglycemic - ≥5.7% HbA1c; Dyslipidemic - >3.4 mmol/L LDL; Low socio-economic - low SES.

AU, arbitrary units; CVD, cardiovascular disease.

**Supplementary Table 2. Additional analysis, metabolomic data of control and cardiovascular disease risk group**

| ***Metabolomic data*** | **Control group (N=166)** | **CVD risk group (N=1036)** | **P-value** | **q-value** |
| --- | --- | --- | --- | --- |
| Ornithine, AU | 8.93 (7.24; 11.0) | 7.46 (6.92; 7.94) | 0.115 | 0.494 |
| 5-Hydroxylysine, AU | 9.23 (7.94; 10.7) | 9.71 (9.12; 10.2) | 0.526 | 0.843 |
| Histidine, AU | 731 (661; 813) | 731 (708; 759) | 0.984 | 0.984 |
| Lysine, AU | 36.1 (31.6; 41.7) | 37.1 (35.5; 38.9) | 0.712 | 0.860 |
| Arginine, AU | 11.8 (10.7; 12.9) | 12.1 (11.8; 12.6) | 0.605 | 0.843 |
| Asparagine, AU | 24.6 (22.4; 27.5) | 24.1 (22.9; 25.1) | 0.709 | 0.860 |
| Glycine, AU | 504 (437; 575) | 526 (501; 550) | 0.563 | 0.843 |
| Serine, AU | 227 (204; 251) | 236 (224; 245) | 0.523 | 0.843 |
| Glutamine, AU | 223 (195; 251) | 235 (224; 245) | 0.472 | 0.837 |
| Isothreonine, AU | 3.50 (2.51; 4.90) | 3.72 (3.31; 4.17) | 0.731 | 0.860 |
| Dimethylglycine, AU | 16.2 (12.9; 20.4) | 15.9 (14.5; 17.4) | 0.879 | 0.902 |
| Beta-alanine, AU | 46.0 (36.3; 58.9) | 51.6 (46.8; 56.2) | 0.387 | 0.767 |
| Threonine, AU | 78.3 (69.2; 89.1) | 81.5 (77.6; 85.1) | 0.554 | 0.843 |
| Hydroxyproline, AU | 3.36 (2.57; 4.37) | 3.51 (3.24; 3.80) | 0.752 | 0.860 |
| Alanine, AU | 226 (200; 257) | 249 (234; 263) | 0.179 | 0.499 |
| Citrulline, AU | 4.66 (4.07; 5.25) | 4.72 (4.47; 4.90) | 0.854 | 0.900 |
| GABA, AU | 0.25 (0.22; 0.27) | 0.26 (0.25; 0.27) | 0.404 | 0.767 |
| Creatine, AU | 16.6 (14.5; 19.1) | 19.3 (18.2; 20.4) | **0.043** | 0.494 |
| Proline, AU | 1.73 (1.58; 1.91) | 1.83 (1.78; 1.91) | 0.344 | 0.765 |
| Cystine, AU | 0.61 (0.54; 0.68) | 0.67 (0.65; 0.69) | 0.129 | 0.494 |
| Valine, AU | 4.73 (4.27; 5.25) | 5.20 (5.01; 5.37) | 0.111 | 0.494 |
| Methionine, AU | 1.14 (1.02; 1.26) | 1.20 (1.15; 1.26) | 0.353 | 0.765 |
| Tyrosine, AU | 32.4 (28.8; 36.3) | 37.8 (36.3; 39.8) | **0.009** | 0.351 |
| Pyroglutamic acid, AU | 23.3 (20.9; 25.7) | 24.8 (24.0; 25.7) | 0.285 | 0.707 |
| Leucine/Isoleucine, AU | 12.0 (10.7; 13.5) | 13.2 (12.6; 13.8) | 0.120 | 0.494 |
| Phenylalanine, AU | 16.3 (14.5; 18.2) | 18.4 (17.8; 19.1) | 0.059 | 0.494 |
| Aspartic acid, AU | 4.58 (4.17; 5.01) | 4.78 (4.57; 4.90) | 0.413 | 0.767 |
| Tryptophan, AU | 1.13 (0.98; 1.29) | 1.30 (1.23; 1.38) | 0.064 | 0.494 |
| Glutamic acid, AU | 2.17 (1.95; 2.45) | 2.37 (2.29; 2.45) | 0.150 | 0.494 |
| 2-Aminoadipic acid, AU | 0.87 (0.76; 1.00) | 0.89 (0.83; 0.93) | 0.805 | 0.872 |
| Free carnitine, AU | 45.3 (40.7; 50.1) | 46.8 (44.7; 49.0) | 0.586 | 0.843 |
| Acetylcarnitine, AU | 11.9 (9.55; 14.8) | 11.4 (10.5; 12.3) | 0.719 | 0.860 |
| Propionylcarnitine, AU | 1.65 (1.35; 2.00) | 1.70 (1.58; 1.82) | 0.772 | 0.860 |
| Butyrylcarnitine, AU | 11.5 (10.0; 13.2) | 11.2 (10.7; 11.8) | 0.722 | 0.860 |
| Isovalerylcarnitine, AU | 0.51 (0.45; 0.59) | 0.58 (0.55; 0.60) | 0.152 | 0.494 |
| Hexanoylcarnitine, AU | 0.06 (0.05; 0.07) | 0.07 (0.06; 0.07) | 0.175 | 0.499 |
| Octanoylcarnitine, AU | 0.31 (0.28; 0.34) | 0.34 (0.32; 0.35) | 0.099 | 0.494 |
| Decanoylcarnitine, AU | 0.18 (0.16; 0.21) | 0.21 (0.19; 0.22) | 0.107 | 0.494 |
| Dodecanoylcarnitine, AU | 0.02 (0.02; 0.03) | 0.02 (0.02; 0.03) | 0.290 | 0.707 |

Test used: ANCOVAs (adjusted for sex, ethnicity, and protein intake). Data are presented as geometric mean with 95% confidence intervals. Benjamini-Hochberg correction (q-value). Bold values denote P/q≤0.05. Cardiovascular disease risk group criteria: Obese - ≥0.55 waist-to-height ratio; Physically inactive - <600 METs for moderate and/or vigorous intensity physical activity; Smoking - ≥11 ng/mL cotinine & self-reported smoking; Excessive alcohol intake - ≥49 U/L GGT & self-reported drinking; Masked hypertensive - normal clinic BP & 24h/day/night BP classified as hypertensive; Hyperglycemic - ≥5.7% HbA1c; Dyslipidemic - >3.4 mmol/L LDL; Low socio-economic - low SES.

AU, arbitrary units; CVD, cardiovascular disease.

**Supplementary Table 3A. Multi-variable adjusted regression analysis with central systolic blood pressure or pulse wave velocity as the dependent variable, with the metabolomics data in the control and cardiovascular disease risk group**

|  | **Central systolic BP, mmHg** | | | | **Pulse wave velocity, m/sec** | | | |
| --- | --- | --- | --- | --- | --- | --- | --- | --- |
|  | **Control group (N=166)** | | **CVD risk group (N=1036)** | | **Control group (N=166)** | | **CVD risk group (N=1036)** | |
| ***Metabolomic data*** | **Adj R^2^** | **β (95%Cl)** | **Adj R^2^** | **β (95%Cl)** | **Adj R^2^** | **β (95%Cl)** | **Adj R^2^** | **β (95%Cl)** |
| Ornithine, AU | **0.20*** | -0.12 (-0.39; 0.14) | **0.11**** | -0.04 (-0.13; 0.06) | **0.32**** | 0.13 (-0.11; 0.37) | **0.27**** | -0.05 (-0.14; 0.04) |
| Age, years |  | 0.11 (-0.15; 0.39) |  | **0.16 (0.06; 0.24)**** |  | 0.21 (-0.04; 0.45) |  | **0.16 (0.07; 0.25)**** |
| Sex, female/male |  | **0.34 (0.08; 0.61)*** |  | **0.24 (0.15; 0.33)**** |  | **0.30 (0.04; 0.56)*** |  | **0.28 (0.19; 0.38)**** |
| Ethnicity, black/white |  | **-0.28 (-0.62; -0.02)*** |  | **-0.19 (-0.28; -0.10)**** |  | -0.01 (-0.27; 0.26) |  | -0.01 (-0.10; 0.07) |
| MAP, mmHg |  |  |  |  |  | **0.41 (0.14; 0.69)*** |  | **0.32 (0.22; 0.41)**** |
| 5-Hydroxylysine, AU | **0.23**** | -0.07 (-0.24; 0.09) | **0.11**** | -0.01 (-0.08; 0.06) | **0.35**** | 0.05 (-0.10; 0.20) | **0.28**** | 0.02 (-0.04; 0.08) |
| Age, years |  | 0.15 (-0.02; 0.32) |  | **0.15 (0.08; 0.22)**** |  | **0.18 (0.02; 0.33)*** |  | **0.16 (0.09; 0.22)**** |
| Sex, female/male |  | **0.36 (0.20; 0.54)**** |  | **0.24 (0.16; 0.30)**** |  | **0.29 (0.12; 0.46)**** |  | **0.27 (0.21; 0.34)**** |
| Ethnicity, black/white |  | **-0.29 (-0.53; -0.15)**** |  | **-0.20 (-0.26; -0.13)**** |  | 0.01 (-0.16; 0.18) |  | -0.01 (-0.08; 0.05) |
| MAP, mmHg |  |  |  |  |  | **0.39 (0.22; 0.57)**** |  | **0.32 (0.25; 0.39)**** |
| Histidine, AU | **0.24**** | -0.08 (-0.23; 0.07) | **0.12**** | **-0.07 (-0.13; <0.01)*** | **0.35**** | -0.01 (-0.14; 0.13) | **0.28**** | **-0.09 (-0.15; -0.03)*** |
| Age, years |  | 0.13 (-0.02; 0.28) |  | **0.15 (0.09; 0.21)**** |  | **0.18 (0.05; 0.32)*** |  | **0.15 (0.10; 0.21)**** |
| Sex, female/male |  | **0.37 (0.22; 0.53)**** |  | **0.25 (0.18; 0.31)**** |  | **0.30 (0.15; 0.45)**** |  | **0.29 (0.23; 0.35)**** |
| Ethnicity, black/white |  | **-0.30 (-0.51; -0.17)**** |  | **-0.21 (-0.27; -0.14)**** |  | 0.01 (-0.14; 0.17) |  | -0.03 (-0.09; 0.03) |
| MAP, mmHg |  |  |  |  |  | **0.37 (0.22; 0.54)**** |  | **0.32 (0.26; 0.38)**** |
| Lysine, AU | **0.24**** | -0.12 (-0.26; 0.03) | **0.11**** | -0.02 (-0.08; 0.04) | **0.35**** | -0.01 (-0.14; 0.12) | **0.28**** | **-0.06 (-0.11; <0.01)*** |
| Age, years |  | 0.13 (-0.02; 0.29) |  | **0.15 (0.09; 0.21)**** |  | **0.19 (0.05; 0.32)*** |  | **0.15 (0.10; 0.21)**** |
| Sex, female/male |  | **0.35 (0.21; 0.51)**** |  | **0.23 (0.17; 0.29)**** |  | **0.30 (0.15; 0.45)**** |  | **0.27 (0.21; 0.33)**** |
| Ethnicity, black/white |  | **-0.29 (-0.50; -0.16)**** |  | **-0.20 (-0.26; -0.13)**** |  | 0.01 (-0.14; 0.17) |  | -0.01 (-0.07; 0.04) |
| MAP, mmHg |  |  |  |  |  | **0.37 (0.23; 0.53)**** |  | **0.32 (0.26; 0.38)**** |
| Arginine, AU | **0.24**** | -0.12 (-0.29; 0.04) | **0.11**** | **-0.06 (-0.12; <0.01)*** | **0.35**** | -0.04 (-0.18; 0.11) | **0.28**** | -0.05 (-0.10; 0.01) |
| Age, years |  | 0.12 (-0.03; 0.28) |  | **0.15 (0.09; 0.21)**** |  | **0.18 (0.04; 0.32)*** |  | **0.16 (0.10; 0.21)**** |
| Sex, female/male |  | **0.33 (0.19; 0.50)**** |  | **0.22 (0.16; 0.28)**** |  | **0.30 (0.14; 0.44)**** |  | **0.26 (0.20; 0.33)**** |
| Ethnicity, black/white |  | **-0.28 (-0.50; -0.15)**** |  | **-0.20 (-0.26; -0.13)**** |  | 0.02 (-0.13; 0.17) |  | -0.02 (-0.07; 0.04) |
| MAP, mmHg |  |  |  |  |  | **0.37 (0.22; 0.53)**** |  | **0.31 (0.25; 0.38)**** |
| Asparagine, AU | **0.25**** | -0.13 (-0.28; 0.02) | **0.12**** | **-0.09 (-0.15; -0.03)*** | **0.35**** | 0.01 (-0.13; 0.14) | **0.28**** | -0.05 (-0.11; 0.01) |
| Age, years |  | 0.12 (-0.03; 0.27) |  | **0.15 (0.09; 0.21)**** |  | **0.19 (0.05; 0.32)*** |  | **0.15 (0.10; 0.21)**** |
| Sex, female/male |  | **0.35 (0.20; 0.51)**** |  | **0.23 (0.17; 0.29)**** |  | **0.30 (0.15; 0.45)**** |  | **0.27 (0.21; 0.33)**** |
| Ethnicity, black/white |  | **-0.29 (-0.50; -0.16)**** |  | **-0.20 (-0.26; -0.14)**** |  | 0.01 (-0.14; 0.17) |  | -0.02 (-0.08; 0.04) |
| MAP, mmHg |  |  |  |  |  | **0.38 (0.23; 0.54)**** |  | **0.32 (0.25; 0.38)**** |
| Glycine, AU | **0.25**** | -0.14 (-0.30; 0.01) | **0.11**** | -0.04 (-0.10; 0.03) | **0.35**** | -0.06 (-0.20; 0.08) | **0.28**** | -0.04 (-0.09; 0.02) |
| Age, years |  | 0.13 (-0.01; 0.29) |  | **0.15 (0.09; 0.21)**** |  | **0.19 (0.05; 0.32)*** |  | **0.15 (0.10; 0.21)**** |
| Sex, female/male |  | **0.32 (0.17; 0.48)**** |  | **0.23 (0.17; 0.29)**** |  | **0.29 (0.14; 0.44)**** |  | **0.27 (0.21; 0.33)**** |
| Ethnicity, black/white |  | **-0.31 (-0.52; -0.18)**** |  | **-0.20 (-0.26; -0.14)**** |  | 0.01 (-0.14; 0.16) |  | -0.02 (-0.08; 0.04) |
| MAP, mmHg |  |  |  |  |  | **0.36 (0.21; 0.53)**** |  | **0.32 (0.26; 0.38)**** |
| Serine, AU | **0.25**** | -0.12 (-0.28; 0.02) | **0.12**** | **-0.09 (-0.15; -0.03)*** | **0.35**** | -0.01 (-0.15; 0.13) | **0.28**** | -0.05 (-0.11; 0.01) |
| Age, years |  | 0.12 (-0.03; 0.28) |  | **0.14 (0.08; 0.20)**** |  | **0.18 (0.05; 0.32)*** |  | **0.15 (0.09; 0.21)**** |
| Sex, female/male |  | **0.34 (0.19; 0.50)**** |  | **0.23 (0.17; 0.29)**** |  | **0.30 (0.15; 0.44)**** |  | **0.27 (0.21; 0.33)**** |
| Ethnicity, black/white |  | **-0.30 (-0.52; -0.18)**** |  | **-0.21 (-0.27; -0.15)**** |  | 0.01 (-0.14; 0.16) |  | -0.02 (-0.08; 0.04) |
| MAP, mmHg |  |  |  |  |  | **0.37 (0.22; 0.53)**** |  | **0.31 (0.25; 0.38)**** |
| Glutamine, AU | **0.25**** | -0.12 (-0.27; 0.03) | **0.12**** | **-0.08 (-0.14; -0.02)*** | **0.35**** | -0.04 (-0.17; 0.10) | **0.28**** | -0.05 (-0.11; <0.01) |
| Age, years |  | 0.13 (-0.02; 0.28) |  | **0.15 (0.08; 0.21)**** |  | **0.18 (0.05; 0.32)*** |  | **0.15 (0.10; 0.21)**** |
| Sex, female/male |  | **0.35 (0.21; 0.51)**** |  | **0.24 (0.17; 0.30)**** |  | **0.30 (0.15; 0.45)**** |  | **0.27 (0.21; 0.34)**** |
| Ethnicity, black/white |  | **-0.30 (-0.51; -0.17)**** |  | **-0.21 (-0.27; -0.14)**** |  | 0.01 (-0.14; 0.16) |  | -0.02 (-0.08; 0.03) |
| MAP, mmHg |  |  |  |  |  | **0.37 (0.22; 0.53)**** |  | **0.32 (0.25; 0.38)**** |
| Isothreonine, AU | 0.22 | -0.33 (-0.78; 0.07) | **0.10**** | -0.03 (-0.16; 0.10) | 0.20 | <0.01 (-0.43; 0.44) | **0.27**** | -0.06 (-0.19; 0.06) |
| Age, years |  | 0.15 (-0.24; 0.56) |  | **0.15 (0.02; 0.28)*** |  | 0.19 (-0.21; 0.58) |  | **0.15 (0.03; 0.28)*** |
| Sex, female/male |  | 0.26 (-0.15; 0.68) |  | **0.23 (0.10; 0.36)**** |  | 0.30 (-0.14; 0.73) |  | **0.27 (0.14; 0.40)**** |
| Ethnicity, black/white |  | -0.27 (-0.76; 0.13) |  | **-0.20 (-0.33; -0.06)*** |  | 0.01 (-0.43; 0.45) |  | -0.02 (-0.14; 0.11) |
| MAP, mmHg |  |  |  |  |  | 0.38 (-0.09; 0.85) |  | **0.31 (0.18; 0.45)**** |

Test used: Multiple linear regressions. Data are presented as adjusted R^2^ with β coefficient and 95% confidence intervals. Central systolic BP, adjusted for age, sex, ethnicity; pulse wave velocity, adjusted for age, sex, ethnicity, mean arterial pressure. Bold values denote P≤0.05; *P≤0.05; **P≤0.001. Cardiovascular disease risk group criteria: Obese - ≥0.55 waist-to-height ratio; Physically inactive - <600 METs for moderate and/or vigorous intensity physical activity; Smoking - ≥11 ng/mL cotinine & self-reported smoking; Excessive alcohol intake - ≥49 U/L GGT & self-reported drinking; Masked hypertensive - normal clinic BP & 24h/day/night BP classified as hypertensive; Hyperglycemic - ≥5.7% HbA1c; Dyslipidemic - >3.4 mmol/L LDL; Low socio-economic - low SES.

AU, arbitrary units; MAP, mean arterial pressure; BP, blood pressure; CVD, cardiovascular disease.

**Supplementary Table 3B. Multi-variable adjusted regression analysis with central systolic blood pressure or pulse wave velocity as the dependent variable, with the metabolomics data in the control and cardiovascular disease risk group**

|  | **Central systolic BP, mmHg** | | | | **Pulse wave velocity, m/sec** | | | |
| --- | --- | --- | --- | --- | --- | --- | --- | --- |
|  | **Control group (N=166)** | | **CVD risk group (N=1036)** | | **Control group (N=166)** | | **CVD risk group (N=1036)** | |
| ***Metabolomic data*** | **Adj R^2^** | **β (95%Cl)** | **Adj R^2^** | **β (95%Cl)** | **Adj R^2^** | **β (95%Cl)** | **Adj R^2^** | **β (95%Cl)** |
| Dimethylglycine, AU | **0.24**** | -0.08 (-0.24; 0.08) | **0.12**** | **-0.07 (-0.13; -0.01)*** | **0.35**** | -0.07 (-0.21; 0.07) | **0.28**** | -0.01 (-0.07; 0.05) |
| Age, years |  | 0.12 (-0.03; 0.28) |  | **0.15 (0.09; 0.21)**** |  | **0.17 (0.03; 0.31)*** |  | **0.16 (0.10; 0.21)**** |
| Sex, female/male |  | **0.35 (0.20; 0.51)**** |  | **0.24 (0.17; 0.30)**** |  | **0.30 (0.14; 0.44)**** |  | **0.27 (0.21; 0.34)**** |
| Ethnicity, black/white |  | **-0.29 (-0.51; -0.17)**** |  | **-0.20 (-0.26; -0.14)**** |  | 0.02 (-0.14; 0.17) |  | -0.02 (-0.07; 0.04) |
| MAP, mmHg |  |  |  |  |  | **0.37 (0.22; 0.53)**** |  | **0.32 (0.25; 0.38)**** |
| Beta-alanine, AU | **0.23**** | -0.06 (-0.22; 0.10) | **0.11**** | 0.03 (-0.03; 0.09) | **0.35**** | <0.01 (-0.14; 0.14) | **0.28**** | -0.03 (-0.09; 0.03) |
| Age, years |  | 0.15 (<0.01; 0.31) |  | **0.15 (0.09; 0.21)**** |  | **0.19 (0.05; 0.32)*** |  | **0.15 (0.10; 0.21)**** |
| Sex, female/male |  | **0.36 (0.21; 0.52)**** |  | **0.24 (0.17; 0.30)**** |  | **0.30 (0.15; 0.45)**** |  | **0.27 (0.21; 0.33)**** |
| Ethnicity, black/white |  | **-0.30 (-0.52; -0.18)**** |  | **-0.19 (-0.25; -0.13)**** |  | 0.01 (-0.14; 0.17) |  | -0.02 (-0.08; 0.04) |
| MAP, mmHg |  |  |  |  |  | **0.38 (0.23; 0.54)**** |  | **0.32 (0.26; 0.38)**** |
| Threonine, AU | **0.23**** | -0.06 (-0.21; 0.09) | **0.12**** | **-0.08 (-0.14; -0.02)*** | **0.35**** | -0.02 (-0.16; 0.11) | **0.28**** | **-0.06 (-0.12; <0.01)*** |
| Age, years |  | 0.13 (-0.02; 0.29) |  | **0.15 (0.08; 0.20)**** |  | **0.18 (0.05; 0.32)*** |  | **0.15 (0.09; 0.21)**** |
| Sex, female/male |  | **0.35 (0.20; 0.51)**** |  | **0.23 (0.17; 0.29)**** |  | **0.30 (0.14; 0.44)**** |  | **0.27 (0.21; 0.33)**** |
| Ethnicity, black/white |  | **-0.30 (-0.51; -0.17)**** |  | **-0.20 (-0.26; -0.14)**** |  | 0.01 (-0.14; 0.17) |  | -0.02 (-0.08; 0.04) |
| MAP, mmHg |  |  |  |  |  | **0.37 (0.23; 0.53)**** |  | **0.32 (0.25; 0.38)**** |
| Hydroxyproline, AU | 0.12 | 0.13 (-0.32; 0.62) | **0.1**** | 0.01 (-0.12; 0.13) | 0.21 | -0.10 (-0.57; 0.34) | **0.26**** | -0.02 (-0.13; 0.10) |
| Age, years |  | 0.14 (-0.28; 0.57) |  | **0.15 (0.02; 0.27)*** |  | 0.18 (-0.21; 0.57) |  | **0.16 (0.04; 0.27)*** |
| Sex, female/male |  | 0.38 (-0.04; 0.82) |  | **0.24 (0.11; 0.36)**** |  | 0.30 (-0.13; 0.72) |  | **0.27 (0.15; 0.40)**** |
| Ethnicity, black/white |  | -0.30 (-0.82; 0.13) |  | **-0.19 (-0.32; -0.07)*** |  | 0.01 (-0.43; 0.45) |  | -0.02 (-0.13; 0.10) |
| MAP, mmHg |  |  |  |  |  | 0.34 (-0.12; 0.81) |  | **0.32 (0.19; 0.45)**** |
| Alanine, AU | **0.23**** | -0.06 (-0.21; 0.09) | **0.11**** | -0.05 (-0.11; 0.02) | **0.35**** | -0.01 (-0.14; 0.12) | **0.28**** | -0.05 (-0.11; <0.01) |
| Age, years |  | 0.13 (-0.02; 0.29) |  | **0.15 (0.09; 0.21)**** |  | **0.18 (0.05; 0.32)*** |  | **0.15 (0.10; 0.21)**** |
| Sex, female/male |  | **0.35 (0.21; 0.52)**** |  | **0.24 (0.17; 0.30)**** |  | **0.30 (0.15; 0.45)**** |  | **0.27 (0.21; 0.34)**** |
| Ethnicity, black/white |  | **-0.30 (-0.51; -0.17)**** |  | **-0.20 (-0.26; -0.14)**** |  | 0.01 (-0.14; 0.17) |  | -0.02 (-0.08; 0.04) |
| MAP, mmHg |  |  |  |  |  | **0.37 (0.23; 0.53)**** |  | **0.32 (0.26; 0.38)**** |
| Citrulline, AU | **0.24**** | -0.10 (-0.26; 0.06) | **0.11**** | -0.05 (-0.12; 0.02) | **0.35**** | 0.04 (-0.10; 0.19) | **0.28**** | -0.04 (-0.11; 0.02) |
| Age, years |  | 0.13 (-0.04; 0.30) |  | **0.15 (0.08; 0.22)**** |  | **0.19 (0.04; 0.34)*** |  | **0.16 (0.09; 0.22)**** |
| Sex, female/male |  | **0.34 (0.18; 0.52)**** |  | **0.23 (0.16; 0.30)**** |  | **0.30 (0.13; 0.46)**** |  | **0.27 (0.20; 0.34)**** |
| Ethnicity, black/white |  | **-0.29 (-0.53; -0.15)**** |  | **-0.20 (-0.27; -0.13)**** |  | 0.01 (-0.16; 0.18) |  | -0.02 (-0.08; 0.05) |
| MAP, mmHg |  |  |  |  |  | **0.38 (0.22; 0.56)**** |  | **0.32 (0.25; 0.38)**** |
| GABA, AU | **0.24**** | -0.10 (-0.27; 0.06) | **0.12**** | **-0.07 (-0.14; -0.01)*** | **0.35**** | -0.02 (-0.17; 0.13) | **0.28**** | 0.01 (-0.05; 0.07) |
| Age, years |  | 0.13 (-0.02; 0.30) |  | **0.15 (0.08; 0.21)**** |  | **0.19 (0.04; 0.33)*** |  | **0.16 (0.09; 0.22)**** |
| Sex, female/male |  | **0.33 (0.17; 0.50)**** |  | **0.22 (0.15; 0.28)**** |  | **0.30 (0.13; 0.45)**** |  | **0.27 (0.21; 0.34)**** |
| Ethnicity, black/white |  | **-0.28 (-0.51; -0.14)**** |  | **-0.19 (-0.25; -0.12)**** |  | 0.01 (-0.15; 0.18) |  | -0.02 (-0.08; 0.05) |
| MAP, mmHg |  |  |  |  |  | **0.37 (0.21; 0.54)**** |  | **0.32 (0.25; 0.38)**** |
| Creatine, AU | **0.23**** | -0.03 (-0.19; 0.13) | **0.11**** | <0.01 (-0.06; 0.07) | **0.35**** | -0.01 (-0.15; 0.14) | **0.28**** | -0.06 (-0.11; <0.01) |
| Age, years |  | 0.14 (-0.01; 0.29) |  | **0.15 (0.09; 0.21)**** |  | **0.19 (0.05; 0.32)*** |  | **0.16 (0.10; 0.21)**** |
| Sex, female/male |  | **0.35 (0.20; 0.51)**** |  | **0.24 (0.17; 0.30)**** |  | **0.30 (0.14; 0.45)**** |  | **0.25 (0.19; 0.32)**** |
| Ethnicity, black/white |  | **-0.30 (-0.52; -0.17)**** |  | **-0.20 (-0.26; -0.13)**** |  | 0.01 (-0.14; 0.17) |  | -0.02 (-0.07; 0.04) |
| MAP, mmHg |  |  |  |  |  | **0.38 (0.23; 0.54)**** |  | **0.32 (0.25; 0.38)**** |
| Proline, AU | **0.24**** | -0.11 (-0.29; 0.05) | **0.12**** | **-0.08 (-0.13; -0.01)*** | **0.36**** | -0.09 (-0.24; 0.06) | **0.28**** | -0.03 (-0.09; 0.03) |
| Age, years |  | 0.13 (-0.02; 0.28) |  | **0.15 (0.08; 0.20)**** |  | **0.18 (0.04; 0.31)*** |  | **0.15 (0.10; 0.21)**** |
| Sex, female/male |  | **0.33 (0.19; 0.50)**** |  | **0.22 (0.16; 0.28)**** |  | **0.29 (0.14; 0.43)**** |  | **0.27 (0.21; 0.33)**** |
| Ethnicity, black/white |  | **-0.28 (-0.49; -0.15)**** |  | **-0.19 (-0.25; -0.13)**** |  | 0.03 (-0.12; 0.18) |  | -0.01 (-0.07; 0.04) |
| MAP, mmHg |  |  |  |  |  | **0.37 (0.22; 0.53)**** |  | **0.32 (0.25; 0.38)**** |
| Cystine, AU | **0.23**** | 0.01 (-0.14; 0.16) | **0.11**** | -0.03 (-0.09; 0.03) | **0.35**** | -0.03 (-0.16; 0.10) | **0.28**** | -0.04 (-0.09; 0.02) |
| Age, years |  | 0.13 (-0.02; 0.29) |  | **0.15 (0.09; 0.21)**** |  | **0.19 (0.05; 0.32)*** |  | **0.16 (0.10; 0.21)**** |
| Sex, female/male |  | **0.35 (0.21; 0.52)**** |  | **0.24 (0.17; 0.29)**** |  | **0.30 (0.15; 0.44)**** |  | **0.27 (0.21; 0.33)**** |
| Ethnicity, black/white |  | **-0.30 (-0.52; -0.17)**** |  | **-0.20 (-0.26; -0.13)**** |  | 0.01 (-0.14; 0.17) |  | -0.02 (-0.07; 0.04) |
| MAP, mmHg |  |  |  |  |  | **0.38 (0.23; 0.54)**** |  | **0.32 (0.25; 0.38)**** |

Test used: Multiple linear regressions. Data are presented as adjusted R^2^ with β coefficient and 95% confidence intervals. Central systolic BP, adjusted for age, sex, ethnicity; pulse wave velocity, adjusted for age, sex, ethnicity, mean arterial pressure. Bold values denote P≤0.05; *P≤0.05; **P≤0.001. Cardiovascular disease risk group criteria: Obese - ≥0.55 waist-to-height ratio; Physically inactive - <600 METs for moderate and/or vigorous intensity physical activity; Smoking - ≥11 ng/mL cotinine & self-reported smoking; Excessive alcohol intake - ≥49 U/L GGT & self-reported drinking; Masked hypertensive - normal clinic BP & 24h/day/night BP classified as hypertensive; Hyperglycemic - ≥5.7% HbA1c; Dyslipidemic - >3.4 mmol/L LDL; Low socio-economic - low SES.

AU, arbitrary units; MAP, mean arterial pressure; BP, blood pressure; CVD, cardiovascular disease.

**Supplementary Table 3C. Multi-variable adjusted regression analysis with central systolic blood pressure or pulse wave velocity as the dependent variable, with the metabolomics data in the control and cardiovascular disease risk group**

|  | **Central systolic BP, mmHg** | | | | **Pulse wave velocity, m/sec** | | | |
| --- | --- | --- | --- | --- | --- | --- | --- | --- |
|  | **Control group (N=166)** | | **CVD risk group (N=1036)** | | **Control group (N=166)** | | **CVD risk group (N=1036)** | |
| ***Metabolomic data*** | **Adj R^2^** | **β (95%Cl)** | **Adj R^2^** | **β (95%Cl)** | **Adj R^2^** | **β (95%Cl)** | **Adj R^2^** | **β (95%Cl)** |
| Valine, AU | **0.25**** | -0.13 (-0.31; 0.02) | **0.12**** | **-0.07 (-0.13; -0.01)*** | **0.36**** | -0.09 (-0.24; 0.05) | **0.28**** | **-0.07 (-0.12; -0.01)*** |
| Age, years |  | 0.13 (-0.02; 0.29) |  | **0.15 (0.09; 0.21)**** |  | **0.18 (0.05; 0.32)*** |  | **0.16 (0.10; 0.21)**** |
| Sex, female/male |  | **0.35 (0.21; 0.51)**** |  | **0.23 (0.17; 0.29)**** |  | **0.30 (0.15; 0.45)**** |  | **0.27 (0.21; 0.33)**** |
| Ethnicity, black/white |  | **-0.27 (-0.49; -0.14)**** |  | **-0.19 (-0.25; -0.13)**** |  | 0.03 (-0.12; 0.18) |  | -0.01 (-0.07; 0.05) |
| MAP, mmHg |  |  |  |  |  | **0.37 (0.23; 0.53)**** |  | **0.32 (0.25; 0.38)**** |
| Methionine, AU | **0.24**** | -0.09 (-0.29; 0.06) | **0.12**** | **-0.08 (-0.14; -0.02)*** | **0.35**** | -0.06 (-0.23; 0.08) | **0.28**** | -0.05 (-0.11; 0.01) |
| Age, years |  | 0.13 (-0.01; 0.29) |  | **0.15 (0.09; 0.21)**** |  | **0.19 (0.05; 0.32)*** |  | **0.15 (0.10; 0.21)**** |
| Sex, female/male |  | **0.35 (0.20; 0.51)**** |  | **0.23 (0.17; 0.29)**** |  | **0.30 (0.14; 0.44)**** |  | **0.27 (0.21; 0.33)**** |
| Ethnicity, black/white |  | **-0.30 (-0.51; -0.17)**** |  | **-0.21 (-0.27; -0.14)**** |  | 0.01 (-0.14; 0.17) |  | -0.02 (-0.08; 0.04) |
| MAP, mmHg |  |  |  |  |  | **0.37 (0.22; 0.53)**** |  | **0.32 (0.25; 0.38)**** |
| Tyrosine, AU | **0.24**** | -0.10 (-0.25; 0.05) | **0.11**** | -0.04 (-0.10; 0.02) | **0.35**** | -0.05 (-0.18; 0.09) | **0.28**** | **-0.06 (-0.11; <0.01)*** |
| Age, years |  | 0.13 (-0.02; 0.29) |  | **0.15 (0.09; 0.21)**** |  | **0.18 (0.05; 0.32)*** |  | **0.15 (0.10; 0.21)**** |
| Sex, female/male |  | **0.36 (0.21; 0.52)**** |  | **0.23 (0.17; 0.29)**** |  | **0.30 (0.15; 0.45)**** |  | **0.27 (0.21; 0.33)**** |
| Ethnicity, black/white |  | **-0.28 (-0.49; -0.15)**** |  | **-0.20 (-0.26; -0.13)**** |  | 0.02 (-0.13; 0.18) |  | -0.01 (-0.07; 0.04) |
| MAP, mmHg |  |  |  |  |  | **0.37 (0.23; 0.53)**** |  | **0.32 (0.26; 0.38)**** |
| Pyroglutamic acid, AU | **0.26**** | **-0.17 (-0.34; -0.02)*** | **0.12**** | **-0.10 (-0.15; -0.03)*** | **0.35**** | -0.06 (-0.21; 0.08) | **0.28**** | -0.03 (-0.09; 0.02) |
| Age, years |  | 0.13 (-0.02; 0.28) |  | **0.15 (0.09; 0.21)**** |  | **0.18 (0.05; 0.32)*** |  | **0.15 (0.10; 0.21)**** |
| Sex, female/male |  | **0.35 (0.21; 0.51)**** |  | **0.23 (0.17; 0.29)**** |  | **0.30 (0.15; 0.45)**** |  | **0.27 (0.21; 0.33)**** |
| Ethnicity, black/white |  | **-0.29 (-0.50; -0.16)**** |  | **-0.20 (-0.26; -0.14)**** |  | 0.01 (-0.14; 0.17) |  | -0.02 (-0.07; 0.04) |
| MAP, mmHg |  |  |  |  |  | **0.37 (0.22; 0.53)**** |  | **0.32 (0.25; 0.38)**** |
| Leucine/Isoleucine, AU | **0.25**** | -0.12 (-0.30; 0.03) | **0.11**** | **-0.06 (-0.12; <0.01)*** | **0.35**** | -0.06 (-0.21; 0.08) | **0.28**** | **-0.06 (-0.11; <0.01)*** |
| Age, years |  | 0.13 (-0.02; 0.28) |  | **0.15 (0.09; 0.21)**** |  | **0.18 (0.04; 0.32)*** |  | **0.15 (0.10; 0.21)**** |
| Sex, female/male |  | **0.36 (0.22; 0.52)**** |  | **0.23 (0.17; 0.29)**** |  | **0.30 (0.15; 0.45)**** |  | **0.27 (0.21; 0.33)**** |
| Ethnicity, black/white |  | **-0.28 (-0.49; -0.15)**** |  | **-0.19 (-0.25; -0.13)**** |  | 0.02 (-0.13; 0.18) |  | -0.01 (-0.07; 0.04) |
| MAP, mmHg |  |  |  |  |  | **0.37 (0.23; 0.53)**** |  | **0.32 (0.25; 0.38)**** |
| Phenylalanine, AU | **0.25**** | -0.13 (-0.29; 0.02) | **0.11**** | -0.04 (-0.10; 0.02) | **0.35**** | -0.08 (-0.22; 0.06) | **0.28**** | **-0.06 (-0.11; <0.01)*** |
| Age, years |  | 0.13 (-0.02; 0.29) |  | **0.15 (0.09; 0.21)**** |  | **0.18 (0.05; 0.32)*** |  | **0.15 (0.10; 0.21)**** |
| Sex, female/male |  | **0.36 (0.22; 0.52)**** |  | **0.23 (0.17; 0.29)**** |  | **0.31 (0.15; 0.45)**** |  | **0.27 (0.21; 0.33)**** |
| Ethnicity, black/white |  | **-0.27 (-0.49; -0.14)**** |  | **-0.20 (-0.26; -0.13)**** |  | 0.03 (-0.13; 0.18) |  | -0.02 (-0.07; 0.04) |
| MAP, mmHg |  |  |  |  |  | **0.37 (0.22; 0.53)**** |  | **0.32 (0.26; 0.38)**** |
| Aspartic acid, AU | **0.25**** | -0.13 (-0.33; 0.02) | **0.12**** | **-0.08 (-0.13; -0.01)*** | **0.35**** | -0.05 (-0.21; 0.10) | **0.28**** | -0.06 (-0.11; <0.01) |
| Age, years |  | 0.13 (-0.02; 0.29) |  | **0.15 (0.09; 0.21)**** |  | **0.18 (0.05; 0.32)*** |  | **0.16 (0.10; 0.21)**** |
| Sex, female/male |  | **0.35 (0.21; 0.51)**** |  | **0.23 (0.16; 0.28)**** |  | **0.30 (0.15; 0.45)**** |  | **0.27 (0.21; 0.33)**** |
| Ethnicity, black/white |  | **-0.28 (-0.49; -0.15)**** |  | **-0.20 (-0.26; -0.13)**** |  | 0.02 (-0.13; 0.17) |  | -0.02 (-0.07; 0.04) |
| MAP, mmHg |  |  |  |  |  | **0.37 (0.22; 0.53)**** |  | **0.32 (0.25; 0.38)**** |
| Tryptophan, AU | **0.24**** | -0.11 (-0.27; 0.05) | **0.11**** | -0.04 (-0.10; 0.02) | **0.35**** | -0.04 (-0.18; 0.10) | **0.28**** | **-0.06 (-0.12; -0.01)*** |
| Age, years |  | 0.13 (-0.02; 0.29) |  | **0.15 (0.09; 0.21)**** |  | **0.18 (0.05; 0.32)*** |  | **0.15 (0.10; 0.21)**** |
| Sex, female/male |  | **0.36 (0.22; 0.52)**** |  | **0.23 (0.17; 0.29)**** |  | **0.30 (0.15; 0.45)**** |  | **0.27 (0.21; 0.33)**** |
| Ethnicity, black/white |  | **-0.27 (-0.49; -0.13)**** |  | **-0.19 (-0.25; -0.13)**** |  | 0.02 (-0.13; 0.18) |  | -0.01 (-0.07; 0.05) |
| MAP, mmHg |  |  |  |  |  | **0.38 (0.23; 0.53)**** |  | **0.32 (0.26; 0.38)**** |
| Glutamic acid, AU | **0.26**** | **-0.16 (-0.34; -0.01)*** | **0.12**** | **-0.09 (-0.15; -0.03)*** | **0.35**** | -0.07 (-0.22; 0.08) | **0.28**** | -0.05 (-0.11; <0.01) |
| Age, years |  | 0.13 (-0.02; 0.29) |  | **0.15 (0.09; 0.21)**** |  | **0.18 (0.05; 0.32)*** |  | **0.15 (0.10; 0.21)**** |
| Sex, female/male |  | **0.35 (0.20; 0.50)**** |  | **0.23 (0.16; 0.28)**** |  | **0.30 (0.15; 0.44)**** |  | **0.27 (0.21; 0.33)**** |
| Ethnicity, black/white |  | **-0.28 (-0.49; -0.15)**** |  | **-0.20 (-0.26; -0.14)**** |  | 0.02 (-0.13; 0.17) |  | -0.02 (-0.07; 0.04) |
| MAP, mmHg |  |  |  |  |  | **0.37 (0.22; 0.53)**** |  | **0.31 (0.25; 0.38)**** |
| 2-Aminoadipic acid, AU | **0.24**** | -0.12 (-0.29; 0.03) | **0.11**** | -0.03 (-0.09; 0.03) | **0.36**** | -0.09 (-0.23; 0.06) | **0.28**** | **-0.07 (-0.13; -0.02)*** |
| Age, years |  | 0.14 (-0.01; 0.30) |  | **0.15 (0.09; 0.21)**** |  | **0.19 (0.05; 0.32)*** |  | **0.16 (0.10; 0.22)**** |
| Sex, female/male |  | **0.37 (0.23; 0.53)**** |  | **0.24 (0.17; 0.30)**** |  | **0.31 (0.16; 0.45)**** |  | **0.27 (0.21; 0.34)**** |
| Ethnicity, black/white |  | **-0.27 (-0.48; -0.13)**** |  | **-0.19 (-0.25; -0.13)**** |  | 0.03 (-0.12; 0.19) |  | <0.01 (-0.06; 0.05) |
| MAP, mmHg |  |  |  |  |  | **0.38 (0.23; 0.54)**** |  | **0.32 (0.26; 0.38)**** |

Test used: Multiple linear regressions. Data are presented as adjusted R^2^ with β coefficient and 95% confidence intervals. Central systolic BP, adjusted for age, sex, ethnicity; pulse wave velocity, adjusted for age, sex, ethnicity, mean arterial pressure. Bold values denote P≤0.05; *P≤0.05; **P≤0.001. Cardiovascular disease risk group criteria: Obese - ≥0.55 waist-to-height ratio; Physically inactive - <600 METs for moderate and/or vigorous intensity physical activity; Smoking - ≥11 ng/mL cotinine & self-reported smoking; Excessive alcohol intake - ≥49 U/L GGT & self-reported drinking; Masked hypertensive - normal clinic BP & 24h/day/night BP classified as hypertensive; Hyperglycemic - ≥5.7% HbA1c; Dyslipidemic - >3.4 mmol/L LDL; Low socio-economic - low SES.

AU, arbitrary units; MAP, mean arterial pressure; BP, blood pressure; CVD, cardiovascular disease. **Supplementary Table 3D. Multi-variable adjusted regression analysis with central systolic blood pressure or pulse wave velocity as the dependent variable, with the metabolomics data in the control and cardiovascular disease risk group**

|  | **Central systolic BP, mmHg** | | | | **Pulse wave velocity, m/sec** | | | |
| --- | --- | --- | --- | --- | --- | --- | --- | --- |
|  | **Control group (N=166)** | | **CVD risk group (N=1036)** | | **Control group (N=166)** | | **CVD risk group (N=1036)** | |
| ***Metabolomic data*** | **Adj R^2^** | **β (95%Cl)** | **Adj R^2^** | **β (95%Cl)** | **Adj R^2^** | **β (95%Cl)** | **Adj R^2^** | **β (95%Cl)** |
| Free carnitine, AU | **0.23**** | -0.03 (-0.21; 0.13) | **0.11**** | -0.03 (-0.09; 0.04) | **0.35**** | -0.05 (-0.20; 0.10) | **0.28**** | <0.01 (-0.06; 0.05) |
| Age, years |  | 0.14 (-0.01; 0.30) |  | **0.15 (0.09; 0.21)**** |  | **0.19 (0.05; 0.33)*** |  | **0.16 (0.10; 0.21)**** |
| Sex, female/male |  | **0.36 (0.21; 0.53)**** |  | **0.24 (0.18; 0.30)**** |  | **0.31 (0.16; 0.46)**** |  | **0.27 (0.21; 0.34)**** |
| Ethnicity, black/white |  | **-0.29 (-0.51; -0.15)**** |  | **-0.19 (-0.25; -0.13)**** |  | 0.03 (-0.13; 0.19) |  | -0.01 (-0.07; 0.04) |
| MAP, mmHg |  |  |  |  |  | **0.37 (0.22; 0.53)**** |  | **0.32 (0.26; 0.38)**** |
| Acetylcarnitine, AU | **0.23**** | -0.02 (-0.18; 0.15) | **0.11**** | -0.03 (-0.10; 0.03) | **0.35**** | -0.04 (-0.19; 0.10) | **0.28**** | <0.01 (-0.06; 0.05) |
| Age, years |  | 0.14 (-0.01; 0.29) |  | **0.15 (0.09; 0.21)**** |  | **0.19 (0.05; 0.32)*** |  | **0.16 (0.10; 0.21)**** |
| Sex, female/male |  | **0.35 (0.21; 0.52)**** |  | **0.24 (0.18; 0.30)**** |  | **0.30 (0.15; 0.45)**** |  | **0.27 (0.21; 0.34)**** |
| Ethnicity, black/white |  | **-0.29 (-0.52; -0.16)**** |  | **-0.19 (-0.25; -0.12)**** |  | 0.02 (-0.13; 0.18) |  | -0.01 (-0.07; 0.04) |
| MAP, mmHg |  |  |  |  |  | **0.38 (0.23; 0.53)**** |  | **0.32 (0.26; 0.38)**** |
| Propionylcarnitine, AU | **0.23**** | -0.07 (-0.24; 0.10) | **0.11**** | -0.01 (-0.08; 0.06) | **0.35**** | -0.07 (-0.22; 0.08) | **0.28**** | -0.01 (-0.08; 0.05) |
| Age, years |  | 0.14 (-0.02; 0.30) |  | **0.15 (0.09; 0.21)**** |  | **0.19 (0.05; 0.33)*** |  | **0.16 (0.10; 0.21)**** |
| Sex, female/male |  | **0.37 (0.22; 0.55)**** |  | **0.24 (0.17; 0.30)**** |  | **0.32 (0.16; 0.48)**** |  | **0.28 (0.21; 0.34)**** |
| Ethnicity, black/white |  | **-0.28 (-0.51; -0.14)**** |  | **-0.19 (-0.26; -0.13)**** |  | 0.03 (-0.13; 0.19) |  | -0.01 (-0.07; 0.05) |
| MAP, mmHg |  |  |  |  |  | **0.37 (0.21; 0.53)**** |  | **0.32 (0.25; 0.38)**** |
| Butyrylcarnitine, AU | **0.25**** | -0.13 (-0.29; 0.02) | **0.12**** | **-0.07 (-0.13; <0.01)*** | **0.36**** | -0.09 (-0.22; 0.05) | **0.28**** | -0.04 (-0.10; 0.02) |
| Age, years |  | 0.14 (-0.01; 0.29) |  | **0.15 (0.09; 0.21)**** |  | **0.19 (0.05; 0.32)*** |  | **0.16 (0.10; 0.21)**** |
| Sex, female/male |  | **0.37 (0.23; 0.53)**** |  | **0.24 (0.18; 0.30)**** |  | **0.31 (0.16; 0.46)**** |  | **0.27 (0.22; 0.34)**** |
| Ethnicity, black/white |  | **-0.26 (-0.48; -0.13)**** |  | **-0.19 (-0.25; -0.12)**** |  | 0.03 (-0.12; 0.19) |  | -0.01 (-0.07; 0.05) |
| MAP, mmHg |  |  |  |  |  | **0.37 (0.23; 0.53)**** |  | **0.32 (0.25; 0.38)**** |
| Isovalerylcarnitine, AU | **0.24**** | -0.08 (-0.26; 0.08) | **0.11**** | -0.01 (-0.08; 0.05) | **0.35**** | -0.06 (-0.22; 0.09) | **0.28**** | -0.05 (-0.11; 0.01) |
| Age, years |  | 0.14 (-0.01; 0.30) |  | **0.15 (0.09; 0.21)**** |  | **0.19 (0.05; 0.33)*** |  | **0.16 (0.10; 0.21)**** |
| Sex, female/male |  | **0.37 (0.23; 0.54)**** |  | **0.24 (0.17; 0.30)**** |  | **0.31 (0.16; 0.46)**** |  | **0.28 (0.22; 0.35)**** |
| Ethnicity, black/white |  | **-0.28 (-0.50; -0.14)**** |  | **-0.19 (-0.25; -0.13)**** |  | 0.03 (-0.13; 0.19) |  | <0.01 (-0.06; 0.06) |
| MAP, mmHg |  |  |  |  |  | **0.38 (0.23; 0.54)**** |  | **0.32 (0.26; 0.38)**** |
| Hexanoylcarnitine, AU | **0.24**** | -0.08 (-0.25; 0.09) | **0.11**** | -0.04 (-0.10; 0.02) | **0.35**** | -0.06 (-0.22; 0.08) | **0.28**** | -0.05 (-0.11; 0.01) |
| Age, years |  | 0.14 (-0.01; 0.30) |  | **0.15 (0.09; 0.21)**** |  | **0.19 (0.05; 0.33)*** |  | **0.16 (0.10; 0.21)**** |
| Sex, female/male |  | **0.35 (0.20; 0.51)**** |  | **0.24 (0.17; 0.29)**** |  | **0.30 (0.14; 0.45)**** |  | **0.27 (0.21; 0.34)**** |
| Ethnicity, black/white |  | **-0.28 (-0.50; -0.15)**** |  | **-0.19 (-0.26; -0.13)**** |  | 0.02 (-0.13; 0.19) |  | -0.01 (-0.07; 0.04) |
| MAP, mmHg |  |  |  |  |  | **0.37 (0.22; 0.54)**** |  | **0.31 (0.25; 0.38)**** |
| Octanoylcarnitine, AU | **0.24**** | -0.11 (-0.31; 0.05) | **0.11**** | -0.04 (-0.10; 0.02) | **0.37**** | -0.13 (-0.31; <0.01) | **0.28**** | -0.05 (-0.10; 0.01) |
| Age, years |  | **0.15 (<0.01; 0.31)*** |  | **0.15 (0.09; 0.21)**** |  | **0.20 (0.07; 0.34)*** |  | **0.16 (0.10; 0.21)**** |
| Sex, female/male |  | **0.35 (0.21; 0.51)**** |  | **0.24 (0.18; 0.30)**** |  | **0.30 (0.15; 0.45)**** |  | **0.28 (0.22; 0.34)**** |
| Ethnicity, black/white |  | **-0.27 (-0.49; -0.14)**** |  | **-0.19 (-0.25; -0.13)**** |  | 0.04 (-0.11; 0.20) |  | -0.01 (-0.07; 0.05) |
| MAP, mmHg |  |  |  |  |  | **0.37 (0.22; 0.53)**** |  | **0.32 (0.25; 0.38)**** |
| Decanoylcarnitine, AU | **0.25**** | -0.13 (-0.29; 0.02) | **0.11**** | -0.06 (-0.12; <0.01) | **0.36**** | -0.11 (-0.25; 0.03) | **0.28**** | -0.05 (-0.11; <0.01) |
| Age, years |  | 0.14 (<0.01; 0.30) |  | **0.15 (0.09; 0.21)**** |  | **0.19 (0.06; 0.33)*** |  | **0.15 (0.10; 0.21)**** |
| Sex, female/male |  | **0.37 (0.23; 0.53)**** |  | **0.24 (0.18; 0.30)**** |  | **0.32 (0.16; 0.46)**** |  | **0.28 (0.22; 0.34)**** |
| Ethnicity, black/white |  | **-0.27 (-0.48; -0.14)**** |  | **-0.19 (-0.25; -0.13)**** |  | 0.03 (-0.12; 0.19) |  | -0.01 (-0.07; 0.05) |
| MAP, mmHg |  |  |  |  |  | **0.37 (0.22; 0.53)**** |  | **0.32 (0.25; 0.38)**** |
| Dodecanoylcarnitine, AU | **0.27*** | **-0.32 (-0.66; -0.03)*** | **0.11**** | 0.08 (-0.04; 0.19) | **0.28*** | -0.07 (-0.38; 0.24) | **0.27**** | -0.06 (-0.16; 0.05) |
| Age, years |  | 0.20 (-0.08; 0.51) |  | **0.15 (0.03; 0.26)*** |  | 0.20 (-0.08; 0.49) |  | **0.16 (0.05; 0.27)*** |
| Sex, female/male |  | **0.30 (0.01; 0.59)*** |  | **0.24 (0.12; 0.35)**** |  | 0.30 (-0.01; 0.59) |  | **0.27 (0.16; 0.39)**** |
| Ethnicity, black/white |  | **-0.34 (-0.71; -0.06)*** |  | **-0.20 (-0.31; -0.08)**** |  | <0.01 (-0.31; 0.31) |  | -0.01 (-0.12; 0.09) |
| MAP, mmHg |  |  |  |  |  | **0.35 (0.03; 0.69)*** |  | **0.31 (0.20; 0.43)**** |

Test used: Multiple linear regressions. Data are presented as adjusted R^2^ with β coefficient and 95% confidence intervals. Central systolic BP, adjusted for age, sex, ethnicity; pulse wave velocity, adjusted for age, sex, ethnicity, mean arterial pressure. Bold values denote P≤0.05; *P≤0.05; **P≤0.001. Cardiovascular disease risk group criteria: Obese - ≥0.55 waist-to-height ratio; Physically inactive - <600 METs for moderate and/or vigorous intensity physical activity; Smoking - ≥11 ng/mL cotinine & self-reported smoking; Excessive alcohol intake - ≥49 U/L GGT & self-reported drinking; Masked hypertensive - normal clinic BP & 24h/day/night BP classified as hypertensive; Hyperglycemic - ≥5.7% HbA1c; Dyslipidemic - >3.4 mmol/L LDL; Low socio-economic - low SES.

AU, arbitrary units; MAP, mean arterial pressure; BP, blood pressure; CVD, cardiovascular disease.

**Supplementary Table 4A. Sensitivity analysis with central systolic blood pressure or pulse wave velocity as the dependent variable, with the metabolomics data in the control and cardiovascular disease risk group**

|  | **Central systolic BP, mmHg** | | | | **Pulse wave velocity, m/sec** | | | |
| --- | --- | --- | --- | --- | --- | --- | --- | --- |
|  | **Control group (N=166)** | | **CVD risk group (N=1036)** | | **Control group (N=166)** | | **CVD risk group (N=1036)** | |
| ***Metabolomic data*** | **Adj R^2^** | **β (95%Cl)** | **Adj R^2^** | **β (95%Cl)** | **Adj R^2^** | **β (95%Cl)** | **Adj R^2^** | **β (95%Cl)** |
| Ornithine, AU | **0.19*** | -0.13 (-0.40; 0.14) | **0.11**** | -0.04 (-0.13; 0.06) | **0.31**** | 0.14 (-0.12; 0.38) | **0.27**** | -0.05 (-0.14; 0.04) |
| Age, years |  | 0.10 (-0.18; 0.39) |  | **0.16 (0.06; 0.24)**** |  | 0.22 (-0.03; 0.47) |  | **0.16 (0.07; 0.25)**** |
| Sex, female/male |  | **0.36 (0.08; 0.66)*** |  | **0.23 (0.13; 0.33)**** |  | 0.27 (-0.01; 0.55) |  | **0.28 (0.19; 0.38)**** |
| Ethnicity, black/white |  | -0.26 (-0.62; 0.01) |  | **-0.21 (-0.30; -0.11)**** |  | -0.02 (-0.31; 0.26) |  | -0.01 (-0.10; 0.07) |
| Protein intake, g |  | -0.08 (-0.37; 0.21) |  | 0.04 (-0.05; 0.14) |  | 0.09 (-0.17; 0.34) |  | 0.01 (-0.08; 0.10) |
| MAP, mmHg |  |  |  |  |  | **0.41 (0.13; 0.70)*** |  | **0.32 (0.22; 0.41)**** |
| 5-Hydroxylysine, AU | **0.23**** | -0.07 (-0.24; 0.10) | **0.11**** | -0.01 (-0.08; 0.06) | **0.34**** | 0.05 (-0.11; 0.20) | **0.28**** | 0.02 (-0.04; 0.08) |
| Age, years |  | 0.14 (-0.04; 0.32) |  | **0.15 (0.08; 0.22)**** |  | **0.19 (0.03; 0.35)*** |  | **0.16 (0.09; 0.22)**** |
| Sex, female/male |  | **0.38 (0.21; 0.57)**** |  | **0.22 (0.15; 0.29)**** |  | **0.27 (0.09; 0.44)*** |  | **0.27 (0.20; 0.34)**** |
| Ethnicity, black/white |  | **-0.28 (-0.52; -0.13)**** |  | **-0.21 (-0.28; -0.13)**** |  | <0.01 (-0.18; 0.17) |  | -0.02 (-0.08; 0.05) |
| Protein intake, g |  | -0.07 (-0.25; 0.120) |  | 0.05 (-0.03; 0.12) |  | 0.08 (-0.08; 0.24) |  | 0.01 (-0.06; 0.08) |
| MAP, mmHg |  |  |  |  |  | **0.38 (0.21; 0.57)**** |  | **0.32 (0.25; 0.39)**** |
| Histidine, AU | **0.23**** | -0.08 (-0.24; 0.07) | **0.12**** | **-0.06 (-0.13; <0.01)*** | **0.35**** | -0.01 (-0.15; 0.13) | **0.28**** | **-0.09 (-0.15; -0.03)*** |
| Age, years |  | 0.12 (-0.04; 0.28) |  | **0.15 (0.08; 0.21)**** |  | **0.20 (0.05; 0.34)*** |  | **0.15 (0.09; 0.21)**** |
| Sex, female/male |  | **0.39 (0.23; 0.57)**** |  | **0.24 (0.17; 0.30)**** |  | **0.28 (0.11; 0.44)**** |  | **0.28 (0.22; 0.35)**** |
| Ethnicity, black/white |  | **-0.28 (-0.51; -0.15)**** |  | **-0.22 (-0.28; -0.15)**** |  | <0.01 (-0.16; 0.16) |  | -0.03 (-0.09; 0.03) |
| Protein intake, g |  | -0.07 (-0.23; 0.10) |  | 0.04 (-0.02; 0.11) |  | 0.08 (-0.07; 0.22) |  | 0.01 (-0.05; 0.07) |
| MAP, mmHg |  |  |  |  |  | **0.37 (0.22; 0.53)**** |  | **0.32 (0.26; 0.38)**** |
| Lysine, AU | **0.24**** | -0.11 (-0.26; 0.05) | **0.11**** | -0.02 (-0.08; 0.04) | **0.35**** | -0.03 (-0.17; 0.11) | **0.28**** | **-0.06 (-0.11; <0.01)*** |
| Age, years |  | 0.12 (-0.03; 0.29) |  | **0.15 (0.09; 0.21)**** |  | **0.20 (0.06; 0.34)*** |  | **0.15 (0.10; 0.21)**** |
| Sex, female/male |  | **0.37 (0.21; 0.54)**** |  | **0.22 (0.16; 0.29)**** |  | **0.27 (0.11; 0.43)**** |  | **0.27 (0.21; 0.33)**** |
| Ethnicity, black/white |  | **-0.28 (-0.50; -0.15)**** |  | **-0.21 (-0.27; -0.14)**** |  | <0.01 (-0.16; 0.16) |  | -0.02 (-0.07; 0.04) |
| Protein intake, g |  | -0.04 (-0.21; 0.13) |  | 0.04 (-0.02; 0.11) |  | 0.09 (-0.06; 0.24) |  | 0.01 (-0.05; 0.07) |
| MAP, mmHg |  |  |  |  |  | **0.37 (0.22; 0.53)**** |  | **0.32 (0.26; 0.38)**** |
| Arginine, AU | **0.24**** | -0.11 (-0.29; 0.04) | **0.12**** | -0.06 (-0.12; <0.01) | **0.35**** | -0.04 (-0.19; 0.10) | **0.28**** | -0.05 (-0.10; 0.01) |
| Age, years |  | 0.11 (-0.04; 0.27) |  | **0.15 (0.09; 0.21)**** |  | **0.19 (0.05; 0.33)*** |  | **0.16 (0.10; 0.21)**** |
| Sex, female/male |  | **0.35 (0.20; 0.53)**** |  | **0.21 (0.15; 0.28)**** |  | **0.27 (0.11; 0.42)**** |  | **0.26 (0.20; 0.33)**** |
| Ethnicity, black/white |  | **-0.27 (-0.49; -0.13)**** |  | **-0.21 (-0.27; -0.14)**** |  | <0.01 (-0.16; 0.16) |  | -0.02 (-0.08; 0.04) |
| Protein intake, g |  | -0.06 (-0.22; 0.10) |  | 0.04 (-0.02; 0.11) |  | 0.09 (-0.06; 0.23) |  | 0.01 (-0.05; 0.07) |
| MAP, mmHg |  |  |  |  |  | **0.37 (0.21; 0.53)**** |  | **0.31 (0.25; 0.38)**** |
| Asparagine, AU | **0.25**** | -0.13 (-0.29; 0.02) | **0.12**** | **-0.09 (-0.15; -0.02)*** | **0.35**** | 0.01 (-0.13; 0.14) | **0.28**** | -0.05 (-0.11; 0.01) |
| Age, years |  | 0.11 (-0.05; 0.27) |  | **0.15 (0.09; 0.21)**** |  | **0.20 (0.06; 0.34)*** |  | **0.15 (0.10; 0.21)**** |
| Sex, female/male |  | **0.37 (0.21; 0.54)**** |  | **0.22 (0.16; 0.29)**** |  | **0.28 (0.11; 0.43)**** |  | **0.27 (0.21; 0.34)**** |
| Ethnicity, black/white |  | **-0.28 (-0.50; -0.14)**** |  | **-0.21 (-0.28; -0.15)**** |  | <0.01 (-0.16; 0.15) |  | -0.02 (-0.08; 0.04) |
| Protein intake, g |  | -0.07 (-0.23; 0.09) |  | 0.04 (-0.03; 0.11) |  | 0.08 (-0.07; 0.22) |  | 0.01 (-0.05; 0.07) |
| MAP, mmHg |  |  |  |  |  | **0.37 (0.22; 0.54)**** |  | **0.32 (0.25; 0.38)**** |
| Glycine, AU | **0.25**** | -0.14 (-0.30; 0.01) | **0.11**** | -0.03 (-0.10; 0.03) | **0.35**** | -0.06 (-0.20; 0.08) | **0.28**** | -0.04 (-0.09; 0.02) |
| Age, years |  | 0.12 (-0.03; 0.29) |  | **0.15 (0.09; 0.21)**** |  | **0.20 (0.06; 0.34)*** |  | **0.15 (0.10; 0.21)**** |
| Sex, female/male |  | **0.34 (0.18; 0.52)**** |  | **0.22 (0.15; 0.28)**** |  | **0.27 (0.10; 0.42)**** |  | **0.27 (0.20; 0.33)**** |
| Ethnicity, black/white |  | **-0.29 (-0.52; -0.16)**** |  | **-0.21 (-0.28; -0.15)**** |  | -0.01 (-0.17; 0.15) |  | -0.02 (-0.08; 0.04) |
| Protein intake, g |  | -0.06 (-0.23; 0.10) |  | 0.04 (-0.02; 0.11) |  | 0.08 (-0.06; 0.23) |  | 0.01 (-0.05; 0.07) |
| MAP, mmHg |  |  |  |  |  | **0.36 (0.20; 0.52)**** |  | **0.32 (0.25; 0.38)**** |
| Serine, AU | **0.24**** | -0.13 (-0.29; 0.03) | **0.12**** | **-0.09 (-0.15; -0.03)*** | **0.35**** | -0.01 (-0.15; 0.13) | **0.28**** | -0.05 (-0.11; 0.01) |
| Age, years |  | 0.11 (-0.04; 0.27) |  | **0.14 (0.08; 0.20)**** |  | **0.20 (0.05; 0.34)*** |  | **0.15 (0.09; 0.21)**** |
| Sex, female/male |  | **0.36 (0.21; 0.53)**** |  | **0.22 (0.16; 0.28)**** |  | **0.27 (0.11; 0.43)**** |  | **0.27 (0.21; 0.33)**** |
| Ethnicity, black/white |  | **-0.29 (-0.51; -0.16)**** |  | **-0.22 (-0.28; -0.15)**** |  | <0.01 (-0.16; 0.15) |  | -0.02 (-0.08; 0.04) |
| Protein intake, g |  | -0.07 (-0.23; 0.09) |  | 0.04 (-0.03; 0.10) |  | 0.08 (-0.07; 0.22) |  | 0.01 (-0.06; 0.07) |
| MAP, mmHg |  |  |  |  |  | **0.37 (0.22; 0.53)**** |  | **0.31 (0.25; 0.38)**** |
| Glutamine, AU | **0.24**** | -0.12 (-0.27; 0.03) | **0.12**** | **-0.08 (-0.14; -0.01)*** | **0.35**** | -0.04 (-0.18; 0.09) | **0.28**** | -0.05 (-0.11; <0.01) |
| Age, years |  | 0.12 (-0.03; 0.28) |  | **0.15 (0.08; 0.21)**** |  | **0.19 (0.05; 0.33)*** |  | **0.15 (0.10; 0.21)**** |
| Sex, female/male |  | **0.37 (0.22; 0.54)**** |  | **0.23 (0.16; 0.29)**** |  | **0.28 (0.11; 0.43)**** |  | **0.27 (0.21; 0.34)**** |
| Ethnicity, black/white |  | **-0.29 (-0.51; -0.16)**** |  | **-0.22 (-0.28; -0.15)**** |  | <0.01 (-0.16; 0.15) |  | -0.02 (-0.08; 0.04) |
| Protein intake, g |  | -0.06 (-0.22; 0.10) |  | 0.04 (-0.03; 0.11) |  | 0.08 (-0.06; 0.23) |  | 0.01 (-0.05; 0.07) |
| MAP, mmHg |  |  |  |  |  | **0.37 (0.21; 0.53)**** |  | **0.32 (0.25; 0.38)**** |
| Isothreonine, AU | 0.15 | -0.33 (-0.83; 0.13) | **0.09**** | -0.03 (-0.17; 0.10) | 0.13 | -0.01 (-0.51; 0.48) | **0.26**** | -0.06 (-0.19; 0.06) |
| Age, years |  | 0.15 (-0.29; 0.61) |  | **0.15 (0.01; 0.28)*** |  | 0.20 (-0.24; 0.64) |  | **0.15 (0.03; 0.28)*** |
| Sex, female/male |  | 0.26 (-0.22; 0.76) |  | **0.22 (0.08; 0.36)*** |  | 0.27 (-0.24; 0.77) |  | **0.27 (0.13; 0.41)**** |
| Ethnicity, black/white |  | -0.27 (-0.82; 0.19) |  | **-0.21 (-0.35; -0.06)*** |  | <0.01 (-0.50; 0.49) |  | -0.02 (-0.15; 0.11) |
| Protein intake, g |  | <0.01 (-0.47; 0.47) |  | 0.04 (-0.10; 0.19) |  | 0.08 (-0.38; 0.55) |  | 0.01 (-0.13; 0.14) |
| MAP, mmHg |  |  |  |  |  | 0.37 (-0.15; 0.89) |  | **0.31 (0.18; 0.45)**** |

Test used: Multiple linear regressions. Data are presented as adjusted R^2^ with β coefficient and 95% confidence intervals. Central systolic BP, adjusted for age, sex, ethnicity, protein intake; pulse wave velocity, adjusted for age, sex, ethnicity, protein intake, mean arterial pressure. Bold values denote P≤0.05; *P≤0.05; **P≤0.001. Cardiovascular disease risk group criteria: Obese - ≥0.55 waist-to-height ratio; Physically inactive - <600 METs for moderate and/or vigorous intensity physical activity; Smoking - ≥11 ng/mL cotinine & self-reported smoking; Excessive alcohol intake - ≥49 U/L GGT & self-reported drinking; Masked hypertensive - normal clinic BP & 24h/day/night BP classified as hypertensive; Hyperglycemic - ≥5.7% HbA1c; Dyslipidemic - >3.4 mmol/L LDL; Low socio-economic - low SES.

AU, arbitrary units; MAP, mean arterial pressure; BP, blood pressure; CVD, cardiovascular disease.

**Supplementary Table 4B. Sensitivity analysis with central systolic blood pressure or pulse wave velocity as the dependent variable, with the metabolomics data in the control and cardiovascular disease risk group**

|  | **Central systolic BP, mmHg** | | | | **Pulse wave velocity, m/sec** | | | |
| --- | --- | --- | --- | --- | --- | --- | --- | --- |
|  | **Control group (N=166)** | | **CVD risk group (N=1036)** | | **Control group (N=166)** | | **CVD risk group (N=1036)** | |
| ***Metabolomic data*** | **Adj R^2^** | **β (95%Cl)** | **Adj R^2^** | **β (95%Cl)** | **Adj R^2^** | **β (95%Cl)** | **Adj R^2^** | **β (95%Cl)** |
| Dimethylglycine, AU | **0.24**** | -0.09 (-0.25; 0.07) | **0.12**** | **-0.07 (-0.13; <0.01)*** | **0.35**** | -0.06 (-0.21; 0.08) | **0.28**** | -0.01 (-0.07; 0.05) |
| Age, years |  | 0.11 (-0.05; 0.27) |  | **0.15 (0.09; 0.21)**** |  | **0.18 (0.04; 0.33)*** |  | **0.16 (0.10; 0.21)**** |
| Sex, female/male |  | **0.37 (0.22; 0.55)**** |  | **0.23 (0.16; 0.29)**** |  | **0.28 (0.11; 0.43)**** |  | **0.27 (0.21; 0.34)**** |
| Ethnicity, black/white |  | **-0.28 (-0.50; -0.14)**** |  | **-0.21 (-0.27; -0.14)**** |  | <0.01 (-0.16; 0.16) |  | -0.02 (-0.08; 0.04) |
| Protein intake, g |  | -0.08 (-0.25; 0.08) |  | 0.04 (-0.02; 0.11) |  | 0.07 (-0.08; 0.22) |  | 0.01 (-0.05; 0.07) |
| MAP, mmHg |  |  |  |  |  | **0.37 (0.21; 0.53)**** |  | **0.32 (0.25; 0.38)**** |
| Beta-alanine, AU | **0.23**** | -0.05 (-0.22; 0.11) | **0.11**** | 0.03 (-0.03; 0.09) | **0.35**** | -0.02 (-0.16; 0.13) | **0.28**** | -0.03 (-0.09; 0.03) |
| Age, years |  | 0.14 (-0.02; 0.30) |  | **0.15 (0.09; 0.21)**** |  | **0.20 (0.06; 0.34)*** |  | **0.15 (0.10; 0.21)**** |
| Sex, female/male |  | **0.37 (0.22; 0.55)**** |  | **0.23 (0.16; 0.29)**** |  | **0.28 (0.11; 0.43)**** |  | **0.27 (0.21; 0.33)**** |
| Ethnicity, black/white |  | **-0.29 (-0.51; -0.16)**** |  | **-0.2 (-0.27; -0.14)**** |  | <0.01 (-0.16; 0.15) |  | -0.02 (-0.08; 0.04) |
| Protein intake, g |  | -0.06 (-0.23; 0.11) |  | 0.05 (-0.02; 0.11) |  | 0.08 (-0.07; 0.23) |  | 0.01 (-0.05; 0.07) |
| MAP, mmHg |  |  |  |  |  | **0.37 (0.22; 0.53)**** |  | **0.32 (0.26; 0.38)**** |
| Threonine, AU | **0.23**** | -0.05 (-0.21; 0.10) | **0.12**** | **-0.08 (-0.14; -0.01)*** | **0.35**** | -0.03 (-0.17; 0.11) | **0.28**** | **-0.06 (-0.12; <0.01)*** |
| Age, years |  | 0.12 (-0.03; 0.28) |  | **0.15 (0.08; 0.20)**** |  | **0.19 (0.05; 0.33)*** |  | **0.15 (0.09; 0.21)**** |
| Sex, female/male |  | **0.37 (0.21; 0.54)**** |  | **0.22 (0.16; 0.28)**** |  | **0.27 (0.11; 0.43)**** |  | **0.27 (0.21; 0.33)**** |
| Ethnicity, black/white |  | **-0.28 (-0.51; -0.15)**** |  | **-0.21 (-0.28; -0.15)**** |  | <0.01 (-0.16; 0.16) |  | -0.02 (-0.08; 0.04) |
| Protein intake, g |  | -0.07 (-0.23; 0.10) |  | 0.04 (-0.02; 0.11) |  | 0.08 (-0.06; 0.23) |  | 0.01 (-0.05; 0.07) |
| MAP, mmHg |  |  |  |  |  | **0.37 (0.22; 0.53)**** |  | **0.32 (0.25; 0.38)**** |
| Hydroxyproline, AU | 0.06 | 0.14 (-0.34; 0.67) | **0.10**** | 0.01 (-0.12; 0.14) | 0.16 | -0.12 (-0.61; 0.36) | **0.26**** | -0.02 (-0.13; 0.10) |
| Age, years |  | 0.13 (-0.32; 0.59) |  | **0.15 (0.02; 0.28)*** |  | 0.20 (-0.22; 0.61) |  | **0.16 (0.04; 0.28)*** |
| Sex, female/male |  | 0.41 (-0.07; 0.90) |  | **0.22 (0.09; 0.36)**** |  | 0.27 (-0.21; 0.74) |  | **0.27 (0.14; 0.40)**** |
| Ethnicity, black/white |  | -0.28 (-0.84; 0.19) |  | **-0.21 (-0.34; -0.07)*** |  | -0.01 (-0.48; 0.46) |  | -0.02 (-0.14; 0.11) |
| Protein intake, g |  | -0.09 (-0.56; 0.39) |  | 0.05 (-0.09; 0.18) |  | 0.10 (-0.34; 0.53) |  | 0.01 (-0.12; 0.14) |
| MAP, mmHg |  |  |  |  |  | 0.33 (-0.16; 0.83) |  | **0.32 (0.19; 0.45)**** |
| Alanine, AU | **0.23**** | -0.06 (-0.21; 0.09) | **0.11**** | -0.04 (-0.11; 0.02) | **0.35**** | -0.01 (-0.15; 0.13) | **0.28**** | -0.05 (-0.11; <0.01) |
| Age, years |  | 0.12 (-0.03; 0.28) |  | **0.15 (0.08; 0.21)**** |  | **0.20 (0.05; 0.34)*** |  | **0.15 (0.09; 0.21)**** |
| Sex, female/male |  | **0.38 (0.22; 0.55)**** |  | **0.23 (0.16; 0.29)**** |  | **0.28 (0.11; 0.43)**** |  | **0.27 (0.21; 0.34)**** |
| Ethnicity, black/white |  | **-0.28 (-0.51; -0.15)**** |  | **-0.21 (-0.27; -0.15)**** |  | <0.01 (-0.16; 0.16) |  | -0.02 (-0.08; 0.04) |
| Protein intake, g |  | -0.07 (-0.23; 0.10) |  | 0.04 (-0.02; 0.11) |  | 0.08 (-0.07; 0.22) |  | 0.01 (-0.05; 0.07) |
| MAP, mmHg |  |  |  |  |  | **0.37 (0.22; 0.53)**** |  | **0.32 (0.26; 0.38)**** |
| Citrulline, AU | **0.23**** | -0.10 (-0.26; 0.07) | **0.11**** | -0.05 (-0.12; 0.02) | **0.34**** | 0.04 (-0.11; 0.19) | **0.28**** | -0.04 (-0.11; 0.02) |
| Age, years |  | 0.12 (-0.05; 0.30) |  | **0.15 (0.08; 0.22)**** |  | **0.20 (0.04; 0.35)*** |  | **0.16 (0.09; 0.22)**** |
| Sex, female/male |  | **0.36 (0.18; 0.55)**** |  | **0.22 (0.15; 0.29)**** |  | **0.28 (0.10; 0.45)*** |  | **0.27 (0.20; 0.34)**** |
| Ethnicity, black/white |  | **-0.28 (-0.52; -0.13)**** |  | **-0.21 (-0.28; -0.14)**** |  | <0.01 (-0.18; 0.17) |  | -0.02 (-0.09; 0.05) |
| Protein intake, g |  | -0.07 (-0.25; 0.11) |  | 0.04 (-0.03; 0.12) |  | 0.08 (-0.08; 0.24) |  | 0.01 (-0.06; 0.08) |
| MAP, mmHg |  |  |  |  |  | **0.38 (0.21; 0.56)**** |  | **0.32 (0.25; 0.38)**** |
| GABA, AU | **0.24**** | -0.11 (-0.28; 0.06) | **0.12**** | -0.07 (-0.13; <0.01) | **0.34**** | -0.02 (-0.17; 0.13) | **0.28**** | 0.01 (-0.05; 0.07) |
| Age, years |  | 0.12 (-0.04; 0.29) |  | **0.15 (0.08; 0.21)**** |  | **0.20 (0.05; 0.35)*** |  | **0.16 (0.09; 0.22)**** |
| Sex, female/male |  | **0.35 (0.19; 0.54)**** |  | **0.21 (0.14; 0.28)**** |  | **0.27 (0.10; 0.44)*** |  | **0.27 (0.20; 0.34)**** |
| Ethnicity, black/white |  | **-0.27 (-0.50; -0.12)*** |  | **-0.20 (-0.27; -0.13)**** |  | <0.01 (-0.17; 0.17) |  | -0.02 (-0.08; 0.04) |
| Protein intake, g |  | -0.08 (-0.25; 0.10) |  | 0.04 (-0.04; 0.11) |  | 0.08 (-0.08; 0.23) |  | 0.01 (-0.05; 0.08) |
| MAP, mmHg |  |  |  |  |  | **0.37 (0.21; 0.54)**** |  | **0.32 (0.25; 0.38)**** |
| Creatine, AU | **0.23**** | -0.02 (-0.19; 0.15) | **0.11**** | <0.01 (-0.07; 0.06) | **0.35**** | -0.02 (-0.17; 0.13) | **0.28**** | -0.06 (-0.12; <0.01) |
| Age, years |  | 0.13 (-0.03; 0.29) |  | **0.15 (0.09; 0.21)**** |  | **0.20 (0.06; 0.34)*** |  | **0.16 (0.10; 0.21)**** |
| Sex, female/male |  | **0.37 (0.21; 0.55)**** |  | **0.22 (0.15; 0.29)**** |  | **0.27 (0.10; 0.43)*** |  | **0.25 (0.18; 0.32)**** |
| Ethnicity, black/white |  | **-0.29 (-0.51; -0.15)**** |  | **-0.21 (-0.27; -0.14)**** |  | <0.01 (-0.16; 0.15) |  | -0.02 (-0.08; 0.04) |
| Protein intake, g |  | -0.06 (-0.23; 0.10) |  | 0.05 (-0.02; 0.11) |  | 0.09 (-0.06; 0.23) |  | 0.01 (-0.05; 0.08) |
| MAP, mmHg |  |  |  |  |  | **0.37 (0.22; 0.54)**** |  | **0.32 (0.25; 0.38)**** |
| Proline, AU | **0.24**** | -0.11 (-0.30; 0.05) | **0.12**** | **-0.07 (-0.13; -0.01)*** | **0.36**** | -0.09 (-0.24; 0.06) | **0.28**** | -0.03 (-0.09; 0.03) |
| Age, years |  | 0.12 (-0.04; 0.28) |  | **0.15 (0.08; 0.20)**** |  | **0.19 (0.05; 0.33)*** |  | **0.15 (0.10; 0.21)**** |
| Sex, female/male |  | **0.36 (0.20; 0.53)**** |  | **0.22 (0.15; 0.28)**** |  | **0.26 (0.10; 0.42)**** |  | **0.27 (0.20; 0.33)**** |
| Ethnicity, black/white |  | **-0.26 (-0.49; -0.13)**** |  | **-0.20 (-0.26; -0.14)**** |  | 0.01 (-0.15; 0.17) |  | -0.01 (-0.07; 0.04) |
| Protein intake, g |  | -0.07 (-0.23; 0.09) |  | 0.04 (-0.03; 0.10) |  | 0.08 (-0.07; 0.22) |  | 0.01 (-0.05; 0.07) |
| MAP, mmHg |  |  |  |  |  | **0.36 (0.21; 0.53)**** |  | **0.32 (0.25; 0.38)**** |
| Cystine, AU | **0.23**** | 0.01 (-0.14; 0.17) | **0.11**** | -0.02 (-0.09; 0.04) | **0.35**** | -0.04 (-0.17; 0.09) | **0.28**** | -0.04 (-0.09; 0.02) |
| Age, years |  | 0.12 (-0.03; 0.29) |  | **0.15 (0.09; 0.21)**** |  | **0.20 (0.06; 0.34)*** |  | **0.16 (0.10; 0.21)**** |
| Sex, female/male |  | **0.38 (0.22; 0.55)**** |  | **0.22 (0.16; 0.29)**** |  | **0.27 (0.11; 0.43)**** |  | **0.27 (0.21; 0.34)**** |
| Ethnicity, black/white |  | **-0.29 (-0.51; -0.15)**** |  | **-0.21 (-0.27; -0.14)**** |  | <0.01 (-0.16; 0.16) |  | -0.02 (-0.08; 0.04) |
| Protein intake, g |  | -0.07 (-0.24; 0.10) |  | 0.04 (-0.02; 0.11) |  | 0.09 (-0.06; 0.23) |  | 0.01 (-0.05; 0.07) |
| MAP, mmHg |  |  |  |  |  | **0.37 (0.22; 0.54)**** |  | **0.32 (0.25; 0.38)**** |

Test used: Multiple linear regressions. Data are presented as adjusted R^2^ with β coefficient and 95% confidence intervals. Central systolic BP, adjusted for age, sex, ethnicity, protein intake; pulse wave velocity, adjusted for age, sex, ethnicity, protein intake, mean arterial pressure. Bold values denote P≤0.05; *P≤0.05; **P≤0.001. Cardiovascular disease risk group criteria: Obese - ≥0.55 waist-to-height ratio; Physically inactive - <600 METs for moderate and/or vigorous intensity physical activity; Smoking - ≥11 ng/mL cotinine & self-reported smoking; Excessive alcohol intake - ≥49 U/L GGT & self-reported drinking; Masked hypertensive - normal clinic BP & 24h/day/night BP classified as hypertensive; Hyperglycemic - ≥5.7% HbA1c; Dyslipidemic - >3.4 mmol/L LDL; Low socio-economic - low SES.

AU, arbitrary units; MAP, mean arterial pressure; BP, blood pressure; CVD, cardiovascular disease.

**Supplementary Table 4C. Sensitivity analysis with central systolic blood pressure or pulse wave velocity as the dependent variable, with the metabolomics data in the control and cardiovascular disease risk group**

|  | **Central systolic BP, mmHg** | | | | **Pulse wave velocity, m/sec** | | | |
| --- | --- | --- | --- | --- | --- | --- | --- | --- |
|  | **Control group (N=166)** | | **CVD risk group (N=1036)** | | **Control group (N=166)** | | **CVD risk group (N=1036)** | |
| ***Metabolomic data*** | **Adj R^2^** | **β (95%Cl)** | **Adj R^2^** | **β (95%Cl)** | **Adj R^2^** | **β (95%Cl)** | **Adj R^2^** | **β (95%Cl)** |
| Valine, AU | **0.24**** | -0.13 (-0.31; 0.03) | **0.12**** | **-0.07 (-0.13; -0.01)*** | **0.36**** | -0.11 (-0.27; 0.03) | **0.28**** | **-0.07 (-0.12; -0.01)*** |
| Age, years |  | 0.13 (-0.03; 0.29) |  | **0.15 (0.09; 0.21)**** |  | **0.20 (0.06; 0.34)*** |  | **0.16 (0.10; 0.21)**** |
| Sex, female/male |  | **0.37 (0.21; 0.54)**** |  | **0.22 (0.16; 0.28)**** |  | **0.27 (0.11; 0.42)**** |  | **0.27 (0.21; 0.33)**** |
| Ethnicity, black/white |  | **-0.27 (-0.49; -0.13)**** |  | **-0.20 (-0.26; -0.13)**** |  | 0.01 (-0.14; 0.17) |  | -0.01 (-0.07; 0.05) |
| Protein intake, g |  | -0.04 (-0.21; 0.13) |  | 0.04 (-0.03; 0.11) |  | 0.11 (-0.04; 0.25) |  | 0.01 (-0.06; 0.07) |
| MAP, mmHg |  |  |  |  |  | **0.37 (0.22; 0.53)**** |  | **0.31 (0.25; 0.38)**** |
| Methionine, AU | **0.24**** | -0.09 (-0.28; 0.08) | **0.12**** | **-0.08 (-0.14; -0.01)*** | **0.35**** | -0.07 (-0.24; 0.08) | **0.28**** | -0.05 (-0.11; 0.01) |
| Age, years |  | 0.13 (-0.03; 0.29) |  | **0.15 (0.08; 0.21)**** |  | **0.20 (0.06; 0.34)*** |  | **0.15 (0.10; 0.21)**** |
| Sex, female/male |  | **0.36 (0.21; 0.54)**** |  | **0.22 (0.15; 0.28)**** |  | **0.27 (0.11; 0.42)**** |  | **0.27 (0.21; 0.33)**** |
| Ethnicity, black/white |  | **-0.29 (-0.51; -0.15)**** |  | **-0.22 (-0.28; -0.15)**** |  | <0.01 (-0.16; 0.15) |  | -0.02 (-0.08; 0.04) |
| Protein intake, g |  | -0.06 (-0.22; 0.11) |  | 0.04 (-0.03; 0.10) |  | 0.09 (-0.06; 0.23) |  | <0.01 (-0.06; 0.07) |
| MAP, mmHg |  |  |  |  |  | **0.37 (0.22; 0.53)**** |  | **0.32 (0.25; 0.38)**** |
| Tyrosine, AU | **0.24**** | -0.09 (-0.25; 0.07) | **0.11**** | -0.04 (-0.10; 0.02) | **0.35**** | -0.06 (-0.20; 0.08) | **0.28**** | **-0.06 (-0.11; <0.01)*** |
| Age, years |  | 0.12 (-0.03; 0.29) |  | **0.15 (0.09; 0.21)**** |  | **0.20 (0.06; 0.33)*** |  | **0.15 (0.10; 0.21)**** |
| Sex, female/male |  | **0.37 (0.22; 0.55)**** |  | **0.22 (0.16; 0.29)**** |  | **0.27 (0.11; 0.43)**** |  | **0.27 (0.21; 0.33)**** |
| Ethnicity, black/white |  | **-0.27 (-0.49; -0.13)**** |  | **-0.21 (-0.27; -0.14)**** |  | 0.01 (-0.15; 0.17) |  | -0.02 (-0.08; 0.04) |
| Protein intake, g |  | -0.06 (-0.22; 0.11) |  | 0.04 (-0.02; 0.11) |  | 0.09 (-0.06; 0.23) |  | <0.01 (-0.06; 0.07) |
| MAP, mmHg |  |  |  |  |  | **0.37 (0.22; 0.53)**** |  | **0.32 (0.26; 0.38)**** |
| Pyroglutamic acid, AU | **0.25**** | **-0.16 (-0.34; -0.01)*** | **0.12**** | **-0.09 (-0.15; -0.03)*** | **0.35**** | -0.07 (-0.22; 0.07) | **0.28**** | -0.03 (-0.09; 0.02) |
| Age, years |  | 0.12 (-0.03; 0.28) |  | **0.15 (0.09; 0.21)**** |  | **0.20 (0.06; 0.33)*** |  | **0.15 (0.10; 0.21)**** |
| Sex, female/male |  | **0.37 (0.21; 0.54)**** |  | **0.22 (0.16; 0.28)**** |  | **0.27 (0.11; 0.43)**** |  | **0.27 (0.21; 0.33)**** |
| Ethnicity, black/white |  | **-0.28 (-0.50; -0.15)**** |  | **-0.21 (-0.27; -0.15)**** |  | <0.01 (-0.16; 0.16) |  | -0.02 (-0.08; 0.04) |
| Protein intake, g |  | -0.05 (-0.21; 0.11) |  | 0.04 (-0.03; 0.10) |  | 0.09 (-0.06; 0.23) |  | 0.01 (-0.05; 0.07) |
| MAP, mmHg |  |  |  |  |  | **0.36 (0.21; 0.53)**** |  | **0.32 (0.25; 0.38)**** |
| Leucine/Isoleucine, AU | **0.24**** | -0.12 (-0.30; 0.04) | **0.12**** | -0.06 (-0.12; <0.01) | **0.35**** | -0.08 (-0.23; 0.07) | **0.28**** | **-0.06 (-0.11; <0.01)*** |
| Age, years |  | 0.12 (-0.03; 0.28) |  | **0.15 (0.09; 0.21)**** |  | **0.19 (0.05; 0.33)*** |  | **0.15 (0.10; 0.21)**** |
| Sex, female/male |  | **0.37 (0.22; 0.55)**** |  | **0.22 (0.16; 0.29)**** |  | **0.28 (0.11; 0.43)**** |  | **0.27 (0.21; 0.34)**** |
| Ethnicity, black/white |  | **-0.27 (-0.49; -0.13)**** |  | **-0.20 (-0.27; -0.14)**** |  | 0.01 (-0.15; 0.17) |  | -0.01 (-0.07; 0.05) |
| Protein intake, g |  | -0.05 (-0.21; 0.12) |  | 0.04 (-0.03; 0.11) |  | 0.10 (-0.05; 0.24) |  | 0.01 (-0.06; 0.07) |
| MAP, mmHg |  |  |  |  |  | **0.37 (0.22; 0.53)**** |  | **0.32 (0.25; 0.38)**** |
| Phenylalanine, AU | **0.24**** | -0.12 (-0.29; 0.04) | **0.11**** | -0.04 (-0.10; 0.03) | **0.36**** | -0.10 (-0.24; 0.05) | **0.28**** | **-0.06 (-0.11; <0.01)*** |
| Age, years |  | 0.12 (-0.03; 0.28) |  | **0.15 (0.09; 0.21)**** |  | **0.20 (0.06; 0.33)*** |  | **0.15 (0.10; 0.21)**** |
| Sex, female/male |  | **0.38 (0.22; 0.55)**** |  | **0.22 (0.16; 0.29)**** |  | **0.28 (0.12; 0.43)**** |  | **0.27 (0.21; 0.33)**** |
| Ethnicity, black/white |  | **-0.27 (-0.49; -0.13)**** |  | **-0.21 (-0.27; -0.14)**** |  | 0.01 (-0.15; 0.17) |  | -0.02 (-0.08; 0.04) |
| Protein intake, g |  | -0.05 (-0.21; 0.12) |  | 0.04 (-0.02; 0.11) |  | 0.10 (-0.05; 0.24) |  | <0.01 (-0.06; 0.07) |
| MAP, mmHg |  |  |  |  |  | **0.37 (0.22; 0.53)**** |  | **0.32 (0.26; 0.38)**** |
| Aspartic acid, AU | **0.25**** | -0.13 (-0.33; 0.02) | **0.12**** | **-0.07 (-0.13; -0.01)*** | **0.35**** | -0.05 (-0.22; 0.10) | **0.28**** | -0.05 (-0.11; <0.01) |
| Age, years |  | 0.12 (-0.03; 0.28) |  | **0.15 (0.09; 0.21)**** |  | **0.20 (0.06; 0.34)*** |  | **0.16 (0.10; 0.21)**** |
| Sex, female/male |  | **0.37 (0.22; 0.55)**** |  | **0.22 (0.15; 0.28)**** |  | **0.28 (0.11; 0.43)**** |  | **0.26 (0.20; 0.33)**** |
| Ethnicity, black/white |  | **-0.27 (-0.49; -0.13)**** |  | **-0.21 (-0.27; -0.14)**** |  | <0.01 (-0.15; 0.16) |  | -0.02 (-0.08; 0.04) |
| Protein intake, g |  | -0.06 (-0.22; 0.10) |  | 0.04 (-0.03; 0.11) |  | 0.09 (-0.06; 0.23) |  | 0.01 (-0.05; 0.07) |
| MAP, mmHg |  |  |  |  |  | **0.37 (0.22; 0.53)**** |  | **0.32 (0.25; 0.38)**** |
| Tryptophan, AU | **0.24**** | -0.10 (-0.27; 0.06) | **0.11**** | -0.04 (-0.10; 0.03) | **0.35**** | -0.05 (-0.20; 0.10) | **0.28**** | **-0.06 (-0.12; -0.01)*** |
| Age, years |  | 0.12 (-0.03; 0.29) |  | **0.15 (0.09; 0.21)**** |  | **0.20 (0.06; 0.34)*** |  | **0.15 (0.10; 0.21)**** |
| Sex, female/male |  | **0.38 (0.22; 0.55)**** |  | **0.22 (0.16; 0.28)**** |  | **0.28 (0.11; 0.43)**** |  | **0.27 (0.21; 0.33)**** |
| Ethnicity, black/white |  | **-0.26 (-0.48; -0.12)**** |  | **-0.20 (-0.27; -0.14)**** |  | 0.01 (-0.15; 0.17) |  | -0.01 (-0.07; 0.05) |
| Protein intake, g |  | -0.06 (-0.22; 0.11) |  | 0.04 (-0.02; 0.11) |  | 0.09 (-0.06; 0.23) |  | 0.01 (-0.05; 0.07) |
| MAP, mmHg |  |  |  |  |  | **0.37 (0.22; 0.53)**** |  | **0.32 (0.26; 0.38)**** |
| Glutamic acid, AU | **0.25**** | **-0.16 (-0.34; -0.01)*** | **0.12**** | **-0.09 (-0.15; -0.03)*** | **0.35**** | -0.07 (-0.22; 0.07) | **0.28**** | -0.05 (-0.11; <0.01) |
| Age, years |  | 0.12 (-0.03; 0.28) |  | **0.15 (0.09; 0.21)**** |  | **0.20 (0.06; 0.33)*** |  | **0.15 (0.10; 0.21)**** |
| Sex, female/male |  | **0.36 (0.21; 0.54)**** |  | **0.22 (0.15; 0.28)**** |  | **0.27 (0.11; 0.43)**** |  | **0.27 (0.20; 0.33)**** |
| Ethnicity, black/white |  | **-0.27 (-0.49; -0.14)**** |  | **-0.21 (-0.27; -0.14)**** |  | <0.01 (-0.15; 0.16) |  | -0.02 (-0.08; 0.04) |
| Protein intake, g |  | -0.06 (-0.22; 0.10) |  | 0.04 (-0.03; 0.10) |  | 0.09 (-0.06; 0.23) |  | <0.01 (-0.06; 0.06) |
| MAP, mmHg |  |  |  |  |  | **0.36 (0.21; 0.53)**** |  | **0.31 (0.25; 0.38)**** |
| 2-Aminoadipic acid, AU | **0.24**** | -0.11 (-0.29; 0.05) | **0.11**** | -0.04 (-0.10; 0.03) | **0.36**** | -0.10 (-0.25; 0.04) | **0.28**** | **-0.07 (-0.13; -0.02)*** |
| Age, years |  | 0.13 (-0.02; 0.30) |  | **0.15 (0.09; 0.21)**** |  | **0.20 (0.06; 0.34)*** |  | **0.16 (0.10; 0.22)**** |
| Sex, female/male |  | **0.38 (0.23; 0.56)**** |  | **0.23 (0.16; 0.29)**** |  | **0.28 (0.12; 0.43)**** |  | **0.27 (0.21; 0.34)**** |
| Ethnicity, black/white |  | **-0.26 (-0.48; -0.12)**** |  | **-0.20 (-0.27; -0.14)**** |  | 0.02 (-0.14; 0.18) |  | -0.01 (-0.07; 0.05) |
| Protein intake, g |  | -0.05 (-0.21; 0.12) |  | 0.05 (-0.02; 0.11) |  | 0.10 (-0.05; 0.24) |  | 0.01 (-0.05; 0.07) |
| MAP, mmHg |  |  |  |  |  | **0.37 (0.22; 0.54)**** |  | **0.32 (0.26; 0.38)**** |

Test used: Multiple linear regressions. Data are presented as adjusted R^2^ with β coefficient and 95% confidence intervals. Central systolic BP, adjusted for age, sex, ethnicity, protein intake; pulse wave velocity, adjusted for age, sex, ethnicity, protein intake, mean arterial pressure. Bold values denote P≤0.05; *P≤0.05; **P≤0.001. Cardiovascular disease risk group criteria: Obese - ≥0.55 waist-to-height ratio; Physically inactive - <600 METs for moderate and/or vigorous intensity physical activity; Smoking - ≥11 ng/mL cotinine & self-reported smoking; Excessive alcohol intake - ≥49 U/L GGT & self-reported drinking; Masked hypertensive - normal clinic BP & 24h/day/night BP classified as hypertensive; Hyperglycemic - ≥5.7% HbA1c; Dyslipidemic - >3.4 mmol/L LDL; Low socio-economic - low SES.

AU, arbitrary units; MAP, mean arterial pressure; BP, blood pressure; CVD, cardiovascular disease.

**Supplementary Table 4D. Sensitivity analysis with central systolic blood pressure or pulse wave velocity as the dependent variable, with the metabolomics data in the control and cardiovascular disease risk group**

|  | **Central systolic BP, mmHg** | | | | **Pulse wave velocity, m/sec** | | | |
| --- | --- | --- | --- | --- | --- | --- | --- | --- |
|  | **Control group (N=166)** | | **CVD risk group (N=1036)** | | **Control group (N=166)** | | **CVD risk group (N=1036)** | |
| ***Metabolomic data*** | **Adj R^2^** | **β (95%Cl)** | **Adj R^2^** | **β (95%Cl)** | **Adj R^2^** | **β (95%Cl)** | **Adj R^2^** | **β (95%Cl)** |
| Free carnitine, AU | **0.23**** | -0.02 (-0.20; 0.15) | **0.11**** | -0.03 (-0.09; 0.04) | **0.35**** | -0.06 (-0.22; 0.09) | **0.28**** | <0.01 (-0.06; 0.05) |
| Age, years |  | 0.13 (-0.03; 0.29) |  | **0.15 (0.09; 0.21)**** |  | **0.20 (0.06; 0.34)*** |  | **0.16 (0.10; 0.21)**** |
| Sex, female/male |  | **0.38 (0.22; 0.56)**** |  | **0.23 (0.16; 0.29)**** |  | **0.29 (0.12; 0.44)**** |  | **0.27 (0.21; 0.34)**** |
| Ethnicity, black/white |  | **-0.28 (-0.51; -0.14)**** |  | **-0.20 (-0.27; -0.14)**** |  | 0.01 (-0.15; 0.18) |  | -0.02 (-0.08; 0.04) |
| Protein intake, g |  | -0.06 (-0.23; 0.10) |  | 0.05 (-0.02; 0.11) |  | 0.09 (-0.06; 0.24) |  | 0.01 (-0.05; 0.07) |
| MAP, mmHg |  |  |  |  |  | **0.37 (0.22; 0.53)**** |  | **0.32 (0.26; 0.38)**** |
| Acetylcarnitine, AU | **0.23**** | -0.01 (-0.18; 0.16) | **0.11**** | -0.03 (-0.10; 0.03) | **0.35**** | -0.05 (-0.20; 0.10) | **0.28**** | <0.01 (-0.06; 0.05) |
| Age, years |  | 0.13 (-0.03; 0.29) |  | **0.15 (0.09; 0.21)**** |  | **0.20 (0.06; 0.34)*** |  | **0.16 (0.10; 0.21)**** |
| Sex, female/male |  | **0.37 (0.22; 0.55)**** |  | **0.23 (0.16; 0.29)**** |  | **0.28 (0.11; 0.43)**** |  | **0.27 (0.21; 0.34)**** |
| Ethnicity, black/white |  | **-0.28 (-0.51; -0.14)**** |  | **-0.20 (-0.26; -0.13)**** |  | 0.01 (-0.15; 0.17) |  | -0.02 (-0.08; 0.04) |
| Protein intake, g |  | -0.07 (-0.23; 0.10) |  | 0.05 (-0.02; 0.11) |  | 0.09 (-0.06; 0.23) |  | 0.01 (-0.05; 0.07) |
| MAP, mmHg |  |  |  |  |  | **0.37 (0.22; 0.53)**** |  | **0.32 (0.26; 0.38)**** |
| Propionylcarnitine, AU | **0.23**** | -0.06 (-0.24; 0.11) | **0.11**** | -0.01 (-0.08; 0.05) | **0.35**** | -0.07 (-0.23; 0.08) | **0.28**** | -0.02 (-0.08; 0.05) |
| Age, years |  | 0.13 (-0.03; 0.29) |  | **0.15 (0.08; 0.21)**** |  | **0.20 (0.05; 0.34)*** |  | **0.16 (0.10; 0.22)**** |
| Sex, female/male |  | **0.39 (0.23; 0.58)**** |  | **0.23 (0.16; 0.29)**** |  | **0.30 (0.12; 0.46)**** |  | **0.27 (0.21; 0.34)**** |
| Ethnicity, black/white |  | **-0.27 (-0.50; -0.13)**** |  | **-0.21 (-0.27; -0.14)**** |  | 0.01 (-0.15; 0.18) |  | -0.01 (-0.08; 0.05) |
| Protein intake, g |  | -0.06 (-0.23; 0.10) |  | 0.05 (-0.02; 0.12) |  | 0.09 (-0.07; 0.23) |  | 0.01 (-0.05; 0.07) |
| MAP, mmHg |  |  |  |  |  | **0.36 (0.20; 0.53)**** |  | **0.32 (0.25; 0.38)**** |
| Butyrylcarnitine, AU | **0.24**** | -0.13 (-0.29; 0.03) | **0.12**** | **-0.07 (-0.13; -0.01)*** | **0.36**** | -0.10 (-0.24; 0.04) | **0.28**** | -0.04 (-0.10; 0.02) |
| Age, years |  | 0.13 (-0.02; 0.29) |  | **0.15 (0.09; 0.21)**** |  | **0.20 (0.06; 0.34)*** |  | **0.16 (0.10; 0.21)**** |
| Sex, female/male |  | **0.38 (0.23; 0.56)**** |  | **0.23 (0.16; 0.29)**** |  | **0.28 (0.12; 0.44)**** |  | **0.27 (0.21; 0.34)**** |
| Ethnicity, black/white |  | **-0.26 (-0.48; -0.12)**** |  | **-0.2 (-0.26; -0.13)**** |  | 0.02 (-0.14; 0.18) |  | -0.01 (-0.07; 0.05) |
| Protein intake, g |  | -0.04 (-0.21; 0.12) |  | 0.05 (-0.02; 0.11) |  | 0.10 (-0.05; 0.24) |  | 0.01 (-0.05; 0.07) |
| MAP, mmHg |  |  |  |  |  | **0.37 (0.22; 0.53)**** |  | **0.32 (0.25; 0.38)**** |
| Isovalerylcarnitine, AU | **0.23**** | -0.07 (-0.26; 0.10) | **0.11**** | -0.01 (-0.08; 0.05) | **0.35**** | -0.08 (-0.24; 0.08) | **0.28**** | -0.05 (-0.11; 0.01) |
| Age, years |  | 0.13 (-0.02; 0.30) |  | **0.15 (0.09; 0.21)**** |  | **0.21 (0.07; 0.35)*** |  | **0.16 (0.10; 0.21)**** |
| Sex, female/male |  | **0.39 (0.23; 0.57)**** |  | **0.23 (0.16; 0.29)**** |  | **0.29 (0.13; 0.45)**** |  | **0.28 (0.22; 0.35)**** |
| Ethnicity, black/white |  | **-0.27 (-0.49; -0.13)**** |  | **-0.20 (-0.27; -0.14)**** |  | 0.02 (-0.14; 0.18) |  | <0.01 (-0.07; 0.06) |
| Protein intake, g |  | -0.05 (-0.22; 0.11) |  | 0.05 (-0.02; 0.11) |  | 0.10 (-0.05; 0.24) |  | 0.01 (-0.05; 0.07) |
| MAP, mmHg |  |  |  |  |  | **0.37 (0.22; 0.54)**** |  | **0.32 (0.26; 0.38)**** |
| Hexanoylcarnitine, AU | **0.23**** | -0.08 (-0.26; 0.09) | **0.11**** | -0.04 (-0.10; 0.02) | **0.35**** | -0.06 (-0.22; 0.09) | **0.28**** | -0.05 (-0.11; 0.01) |
| Age, years |  | 0.13 (-0.03; 0.30) |  | **0.15 (0.09; 0.21)**** |  | **0.20 (0.06; 0.34)*** |  | **0.16 (0.10; 0.21)**** |
| Sex, female/male |  | **0.37 (0.21; 0.55)**** |  | **0.22 (0.16; 0.29)**** |  | **0.27 (0.11; 0.43)**** |  | **0.27 (0.21; 0.34)**** |
| Ethnicity, black/white |  | **-0.27 (-0.50; -0.13)**** |  | **-0.21 (-0.27; -0.14)**** |  | 0.01 (-0.15; 0.18) |  | -0.02 (-0.08; 0.04) |
| Protein intake, g |  | -0.07 (-0.24; 0.10) |  | 0.04 (-0.02; 0.11) |  | 0.08 (-0.07; 0.22) |  | 0.01 (-0.06; 0.07) |
| MAP, mmHg |  |  |  |  |  | **0.37 (0.21; 0.54)**** |  | **0.31 (0.25; 0.38)**** |
| Octanoylcarnitine, AU | **0.24**** | -0.11 (-0.32; 0.05) | **0.11**** | -0.04 (-0.10; 0.02) | **0.37**** | -0.14 (-0.32; <0.01) | **0.28**** | -0.05 (-0.10; 0.01) |
| Age, years |  | 0.14 (-0.01; 0.30) |  | **0.15 (0.09; 0.21)**** |  | **0.22 (0.08; 0.35)*** |  | **0.16 (0.10; 0.21)**** |
| Sex, female/male |  | **0.37 (0.22; 0.55)**** |  | **0.23 (0.16; 0.29)**** |  | **0.28 (0.12; 0.43)**** |  | **0.28 (0.21; 0.34)**** |
| Ethnicity, black/white |  | **-0.26 (-0.48; -0.12)**** |  | **-0.20 (-0.27; -0.14)**** |  | 0.03 (-0.13; 0.19) |  | -0.01 (-0.07; 0.05) |
| Protein intake, g |  | -0.06 (-0.23; 0.10) |  | 0.04 (-0.02; 0.11) |  | 0.09 (-0.06; 0.23) |  | 0.01 (-0.06; 0.07) |
| MAP, mmHg |  |  |  |  |  | **0.36 (0.21; 0.53)**** |  | **0.32 (0.25; 0.38)**** |
| Decanoylcarnitine, AU | **0.24**** | -0.13 (-0.29; 0.03) | **0.11**** | -0.06 (-0.12; 0.01) | **0.36**** | -0.12 (-0.26; 0.02) | **0.28**** | -0.05 (-0.11; <0.01) |
| Age, years |  | 0.13 (-0.02; 0.30) |  | **0.15 (0.09; 0.21)**** |  | **0.21 (0.07; 0.34)*** |  | **0.15 (0.10; 0.21)**** |
| Sex, female/male |  | **0.39 (0.23; 0.56)**** |  | **0.23 (0.17; 0.29)**** |  | **0.29 (0.13; 0.44)**** |  | **0.28 (0.22; 0.34)**** |
| Ethnicity, black/white |  | **-0.26 (-0.48; -0.12)**** |  | **-0.20 (-0.26; -0.13)**** |  | 0.02 (-0.14; 0.18) |  | -0.01 (-0.07; 0.05) |
| Protein intake, g |  | -0.05 (-0.22; 0.11) |  | 0.04 (-0.02; 0.11) |  | 0.09 (-0.05; 0.24) |  | 0.01 (-0.05; 0.07) |
| MAP, mmHg |  |  |  |  |  | **0.36 (0.21; 0.52)**** |  | **0.31 (0.25; 0.38)**** |
| Dodecanoylcarnitine, AU | **0.25*** | **-0.32 (-0.68; -0.02)*** | **0.11**** | 0.08 (-0.04; 0.20) | **0.27*** | -0.09 (-0.42; 0.23) | **0.27**** | -0.06 (-0.16; 0.05) |
| Age, years |  | 0.20 (-0.10; 0.52) |  | **0.15 (0.03; 0.26)*** |  | 0.22 (-0.08; 0.52) |  | **0.16 (0.05; 0.27)*** |
| Sex, female/male |  | 0.30 (-0.02; 0.63) |  | **0.23 (0.10; 0.35)**** |  | 0.26 (-0.07; 0.59) |  | **0.27 (0.15; 0.39)**** |
| Ethnicity, black/white |  | **-0.34 (-0.73; -0.05)*** |  | **-0.21 (-0.33; -0.09)**** |  | -0.02 (-0.35; 0.31) |  | -0.01 (-0.13; 0.10) |
| Protein intake, g |  | <0.01 (-0.32; 0.32) |  | 0.05 (-0.07; 0.18) |  | 0.10 (-0.20; 0.40) |  | <0.01 (-0.11; 0.12) |
| MAP, mmHg |  |  |  |  |  | **0.34 (0.01; 0.68)*** |  | **0.31 (0.20; 0.43)**** |

Test used: Multiple linear regressions. Data are presented as adjusted R^2^ with β coefficient and 95% confidence intervals. Central systolic BP, adjusted for age, sex, ethnicity, protein intake; pulse wave velocity, adjusted for age, sex, ethnicity, protein intake, mean arterial pressure. Bold values denote P≤0.05; *P≤0.05; **P≤0.001. Cardiovascular disease risk group criteria: Obese - ≥0.55 waist-to-height ratio; Physically inactive - <600 METs for moderate and/or vigorous intensity physical activity; Smoking - ≥11 ng/mL cotinine & self-reported smoking; Excessive alcohol intake - ≥49 U/L GGT & self-reported drinking; Masked hypertensive - normal clinic BP & 24h/day/night BP classified as hypertensive; Hyperglycemic - ≥5.7% HbA1c; Dyslipidemic - >3.4 mmol/L LDL; Low socio-economic - low SES.

AU, arbitrary units; MAP, mean arterial pressure; BP, blood pressure; CVD, cardiovascular disease.
